# Enhanced redox kinetics of Prussian blue analogues for superior electrochemical deionization performance†

**DOI:** 10.1039/d4sc00686k

**Published:** 2024-06-04

**Authors:** Jiabao Li, Ruoxing Wang, Lanlan Han, Tianyi Wang, Zeinhom M. El-Bahy, Yiyong Mai, Chengyin Wang, Yusuke Yamauchi, Xingtao Xu

**Affiliations:** a School of Chemistry and Chemical Engineering, Yangzhou University Yangzhou 225002 Jiangsu China wangcy@yzu.edu.cn; b Chemistry Department, Faculty of Science, Al-Azhar University Nasr City Cairo Egypt; c School of Chemistry and Chemical Engineering, Frontiers Science Center for Transformative Molecules, Key Laboratory of Green and High-End Utilization of Salt Lake Resources (Chinese Academy of Sciences), Shanghai Key Laboratory of Electrical Insulation and Thermal Ageing, Shanghai Jiao Tong University 800 Dongchuan Road Shanghai 200240 China; d Department of Materials Process Engineering, Graduate School of Engineering, Nagoya University Nagoya 464-8603 Japan; e Department of Plant & Environmental New Resources, College of Life Sciences, Kyung Hee University 1732 Deogyeong-daero, Giheung-gu Yongin-si Gyeonggi-do 17104 South Korea; f Australian Institute for Bioengineering and Nanotechnology (AIBN), The University of Queensland Brisbane QLD 4072 Australia y.yamauchi@uq.edu.au; g Marine Science and Technology College, Zhejiang Ocean University Zhoushan 316022 Zhejiang China xingtao.xu@zjou.edu.cn; h National & Local Joint Engineering Research Center for Mineral Salt Deep Utilization, Huaiyin Institute of Technology Huaian 223003 P. R. China

## Abstract

Prussian blue analogues (PBAs), representing the typical faradaic electrode materials for efficient capacitive deionization (CDI) due to their open architecture and high capacity, have been plagued by kinetics issues, leading to insufficient utilization of active sites and poor structure stability. Herein, to address the conflict issue between desalination capacity and stability due to mismatched ionic and electronic kinetics for the PBA-based electrodes, a rational design, including Mn substitution and polypyrrole (ppy) connection, has been proposed for the nickel hexacyanoferrate (Mn–NiHCF/ppy), serving as a model case. Particularly, the theoretical calculation manifests the reduced bandgap and energy barrier for ionic diffusion after Mn substitution, combined with the increased electronic conductivity and integrity through ppy connecting, resulting in enhanced redox kinetics and boosted desalination performance. Specifically, the optimized Mn–NiHCF/ppy demonstrates a remarkable desalination capacity of 51.8 mg g^−1^ at 1.2 V, accompanied by a high charge efficiency of 81%, and excellent cycling stability without obvious degradation up to 50 cycles, outperforming other related materials. Overall, our concept shown herein provides insights into the design of advanced faradaic electrode materials for high-performance CDI.

## Abstract 2

The Mn substitution and structure optimization *via* ppy connection confer enhanced ion/electron migration to NiHCF. This results in rapid redox kinetics, improves electrode integrity, and ultimately ensures high desalination performance.

## Graphical abstract

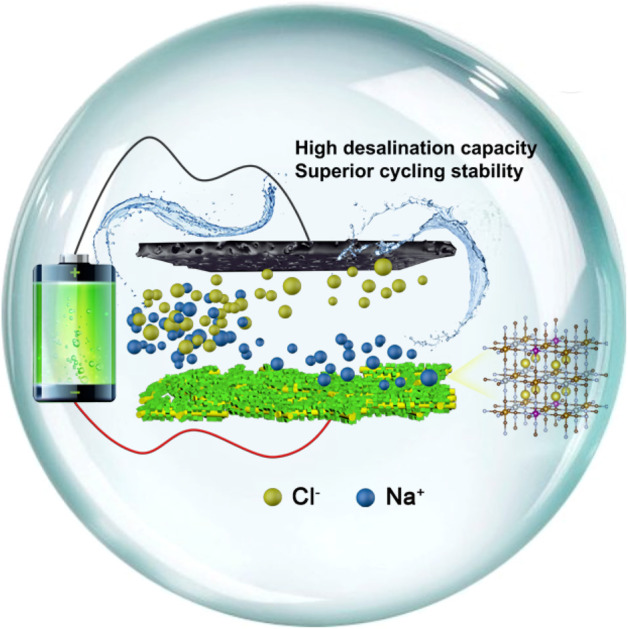

## Introduction

The global freshwater shortage has become one of the most urgent challenges for human society with the rapid development of industry and serious environmental pollution.^1,2^ To alleviate the water crisis, it is necessary to explore and develop novel and low-cost technologies for the desalination of brackish and saline water.^3^ Compared with the conventional desalination technology, such as multistage flash distillation and reverse osmosis, capacitive deionization (CDI), removing ions from saline water based on a pair of electrodes through electrosorption or redox reaction, has been deemed as one of the most promising technologies for desalination based on the advantages of low energy consumption, tuned scale, and mild operating conditions, especially for the low-concentration brines.^4–6^ However, CDI cells based on carbonaceous electrodes often exhibit low desalination capacity and poor cycling life due to their intrinsically low electric double layer (EDL) capacitance, co-ion expulsion effect, and side reactions.^7–10^ Given this, exploring high-performance electrodes, featuring high desalination capacity and good cycling stability, is promising yet challenging for the practical application of CDI.

**Fig. 1:**
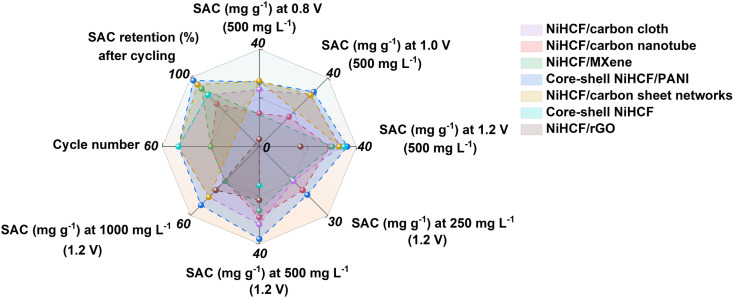
Summary of the NiHCF-based electrodes for CDI reported previously (*e.g.*, NiHCF/carbon cloth;^21^ NiHCF/carbon nanotube;^22^ NiHCF/MXene;^23^ core–shell NiHCF/PANI;^24^ NiHCF/carbon sheet networks;^25^ core–shell NiHCF;^26^ NiHCF/rGO^27^).

Recently, replacing the Na^+^ capture side from the carbonaceous electrode with a faradaic electrode has drawn increasing attention, which involves the redox reaction for CDI process, and dramatically improves the desalination capacity.^11,12^ So far, a broad spectrum of faradaic electrodes has been extensively explored, such as polyanion compounds, metal oxides/sulfides, conductive polymers, MXene, and derivations, *etc.*^13–17^ Noting that the Prussian blue analogues (PBAs) have gained increasing interest for desalination on account of their large spacing inside, open framework, and low insertion/extraction voltage, hence guaranteeing high desalination capacity, modified charge efficiency, and low energy consumption.^11,18–20^ Particularly, such structure advantages render, for example, the nickel hexacyanoferrate (NiHCF) to be a promising electrode candidate with remarkable desalination performance.^11,21^ Promoted by the structure advantages of NiHCF for CDI application, smartly designing the framework of NiHCF and assembling it on a conductive substrate to fabricate a hybrid electrode, aiming to facilitate the desalination performance, has become the mainstream.^11,16,18,20^ However, lacking rational control will cause serious aggregation and stacking of NiHCF upon self-assembly on a conductive substrate, restricting the migration of electrons/ions and inducing high concentration polarization, which in turn degrades the desalination performance.^20^ Based on literature review, the application of NiHCF for CDI, even with modifications of nano-structure design and conductive matrix hybridization, suffers from unsatisfied desalination performance (Fig. 1):^21–27^ (1) limited desalination capacity (usually less than 50 mg g^−1^) due to low electrochemical activity; (2) sluggish desalination rate owing to poor electronic conductivity and intrinsic structural defects; and (3) degraded capacity retention upon cycling (usually less than 90% ≤ 50 cycles) resulting from lattice collapse. Of note, the mismatched kinetics between ions and electrons should be responsible for such degradation on desalination, and more sophisticated structural control and hybridization are required for NiHCF.

To create novel nanomaterials through the self-assembly of building blocks on a conductive substrate is very promising for synthesizing hybrid structures with controlled composition, structure, and morphology.^28^ Specifically, for the atomic-level compositional control, rational substitution at the atomic level for the active material at target sites can dramatically improve electrochemical performance.^29^ For the optimization of structure and morphology, it is crucial not only to achieve the uniform distribution of building blocks on the substrate but also to ensure rational interactions between these building blocks. This is essential for avoiding aggregation and stacking.^30,31^ The comprehensive design of CDI electrode materials, spanning from atomic scale to nanoscale and meso/macroscopic scale, is anticipated to enhance electronic conductivity between the building block and substrate. Simultaneously, improved ionic conductivity among building blocks is expected, reducing the disparity between ionic and electronic kinetics.^30^ This modification in turn enhances redox kinetics and facilitates faradaic reactions.^31^

This work proposes the tailoring of Mn substitution and ppy connection, aiming to achieving rapid redox kinetics and high desalination performance and using NiHCF as the model case (Mn–NiHCF/ppy). The superior electron/ion migration capability of the target Mn–NiHCF/ppy is confirmed through density functional theory (DFT) calculation, combined with systematic characterizations. Additionally, the enhanced desalination performances under different working voltages and feed concentrations have also been demonstrated. As a result, the optimized Mn–NiHCF/ppy achieves a high desalination capacity of 51.8 mg g^−1^ at 1.2 V in a feed solution of 500 mg L^−1^, along with a stable cycling up to 50 cycles and only 0.11% decay per cycle, outperforming other MHCF counterparts.

## Experimental

**Preparation of the Mn–NiHCF/ppy:** according to previous literature,^11,32^ Ni(NO_3_)_2_·6H_2_O (1.0 mmol), Mn(NO_3_)_2_·4H_2_O (0.1 mmol), and sodium citrate (2.25 mmol) were dispersed in deionized water (50 mL) under vigorous stirring to form solution A. Meanwhile, a certain amount of as-prepared ppy (50, 100, and 150 mg) was added into the above solution, respectively. In a separate step, K_3_Fe(CN)_6_ (2.25 mmol) was dispersed in 100 mL of deionized water to form solution B. Afterward, solutions A and B were simultaneously poured into 50 mL of deionized water, which was continuously stirred for 5 hours and then set aside for 24 hours. The resulting mixture was then thoroughly washed with deionized water and dried overnight at 60 °C to obtain the target Mn–NiHCF/ppy. Noting that the samples with the addition of 150, 100, and 50 mg of ppy were denoted as Mn–NiHCF/ppy-1, Mn–NiHCF/ppy-2, and Mn–NiHCF/ppy-3, respectively. For comparison, Mn–NiHCF was synthesized using the same precursor as mentioned above, but without the addition of ppy. Similarly, the pure NiHCH was prepared without the manganese source and ppy in the precursor. Please refer to the ESI† for more details of the preparation of ppy tubes, material characterization, electrochemical testing, and desalination experiments.

## Results and discussion

**Fig. 2:**
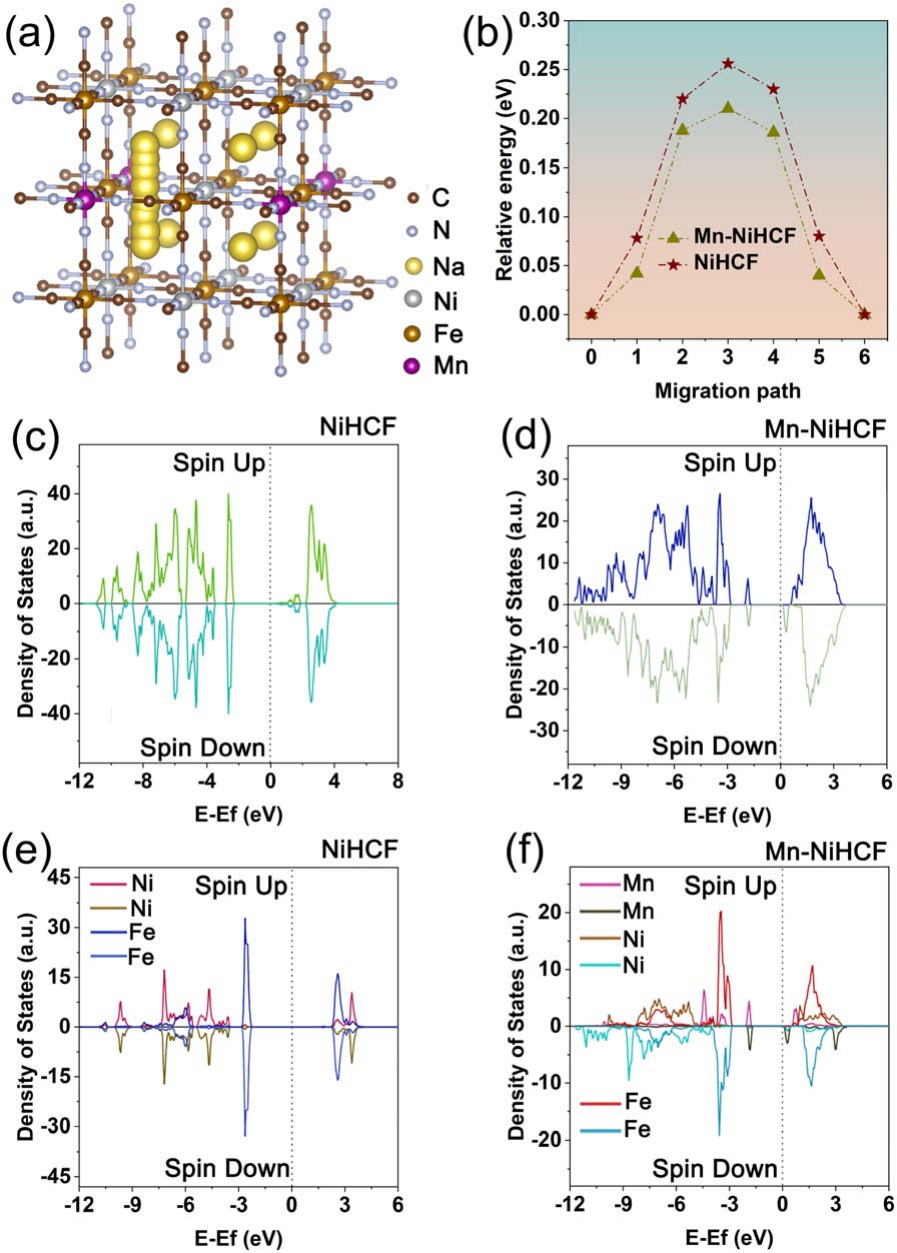
(a) Possible Na^+^ diffusion pathway inside ideal Mn–NiHCF structure; (b) energy barrier profiles of ionic diffusion in NiHCF and Mn–NiHCF; total density of states and partial density of states of (c and e) NiHCF and (d and f) Mn–NiHCF.

Before comparing the discrepancies on ionic kinetics and electronic properties after Mn substitution based on DFT calculation, the optimized substitution sites of Mn in the NiHCF framework should be determined. As shown in Fig. S1 (ESI†), the results reveal that the total energies for Mn substitution at the high-spin Ni sites and low-spin Fe sites are calculated to be −0.32 and −0.23 eV, respectively. This analysis establishes that Mn substitution at the high-spin Ni sites is more stable compared to the low-spin Fe sites. Our findings align with prior studies in the literature, indicating that the preferred substitution site for Mn in MHCFs is the high-spin metal sites.^32–36^ Based on the results of substitution site calculation, the positive effect of Mn substitution for the ionic diffusion and energy barrier in NiHCF and Mn–NiHCF has been compared. The possible diffusion pathway and the corresponding energy barrier for Mn–NiHCF are illustrated in Fig. 2a and b, with the calculated results for NiHCF shown in Fig. S2 (ESI†). It is evident that the energy barrier of the substituted sample is significantly lower than that of pristine NiHCF. The respective values are 0.25 eV for NiHCF and 0.21 eV for Mn–NiHCF. This discrepancy highlights the altered ionic diffusion kinetics attributed to the Mn substitution.^18,37,38^ Apart from the ionic kinetics, the electronic properties of NiHCF and Mn–NiHCF have also been compared through the total density of state (TDOS) and partial density of state (PDOS). From the TDOSs of NiHCF and Mn–NiHCF (Fig. 2c and d), the bandgap of NiHCF (2.45 eV) is reduced after Mn substitution, indicating the modified electronic conductivity for the Mn–NiHCF.^32^ Moreover, a detailed investigation of the PDOSs for the two samples (Fig. 2e and f) reveals that it is the impurity energy levels induced by Mn 3d states, as well as its interaction with the Ni 3d states, lead to the reduction of the bandgap.^39,40^ Of note, the Fermi level almost shifts to the newly formed conduction band for the Mn–NiHCF, further combining with the enhanced ionic diffusion kinetics, which highlights the modified ionic/electronic dynamics after Mn substitution.^32^ Particularly, the Mn substitution shows advantages for increasing the ionic/electronic conductivity of the NiHCF, hence facilitating the redox reaction upon desalination.^32,41^ Given this perspective, the strategic doping of metals, aimed at achieving modified electronic and ionic transfer concurrently, emerges as a promising strategy for altering the internal structure and properties of PBAs.

**Fig. 3:**
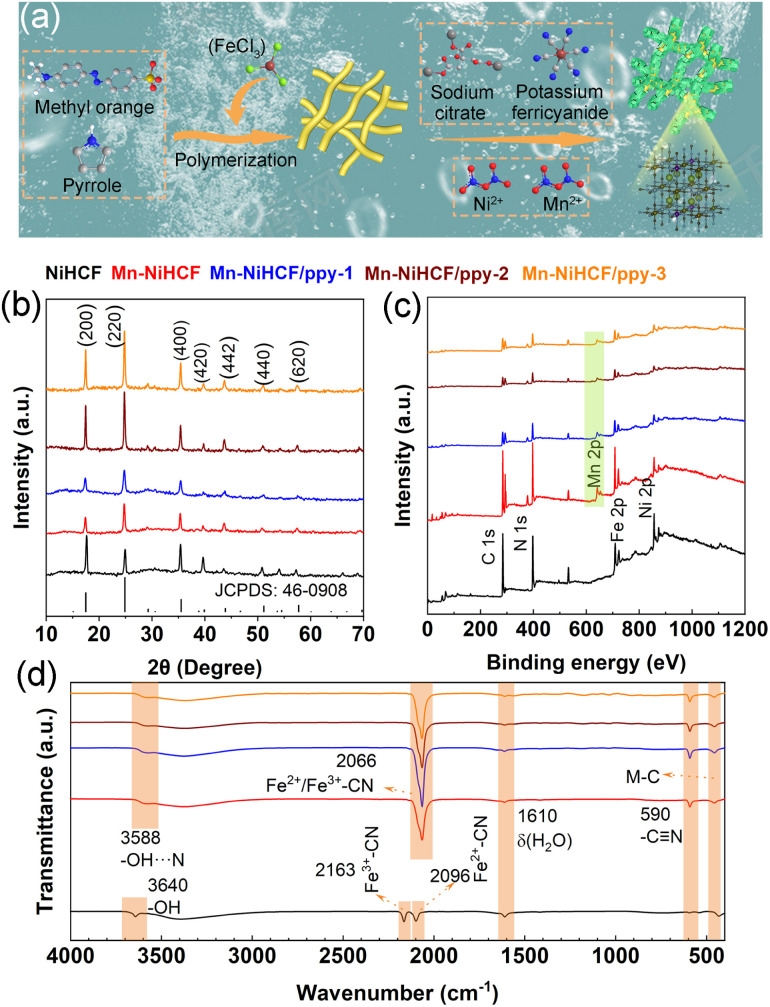
(a) Schematic depiction for the preparation of Mn–NiHCF/ppy; (b) XRD patterns, (c) XPS survey spectra, and (d) FTIR spectra of NiHCF, Mn–NiHCF, Mn–NiHCF/ppy-1, Mn–NiHCF/ppy-2, and Mn–NiHCF/ppy-3.

The DFT calculation results above have proved the superiority of Mn–NiHCF on ionic/electronic kinetics, thus verifying the significance of rational Mn substitution for NiHCF. To get a clear understanding for the synthesis of the target Mn–NiHCF/ppy, the schematic illustration has been shown in Fig. 3a. First of all, the tubular ppy was fabricated through a reported method, employing the ferric trichloride as the initiator.^42^ Due to its nitrogen-containing functional groups and electronegativity in solution, the ppy tubes can be utilized as an ideal substrate for the *in situ* growth of Mn–NiHCF, where the addition of sodium citrate, potassium ferricyanide, Ni(NO_3_)_2_·6H_2_O, and Mn(NO_3_)_2_·4H_2_O with stoichiometric ratio thus results in the crystallization of Mn–NiHCF on ppy.^43–45^Fig. 3b depicts the X-ray diffraction (XRD) patterns of the as-prepared samples. As shown, the diffraction peaks for the five samples can be well indexed to the JCPDS no. 46-0908, thus verifying the Mn substitution and introduction of ppy show little effect for the Prussian blue crystal structure.^7,16^ Notably, the closed investigation for the (200) peak at around 17.6° reveals that the Mn substitution leads to its left shift (Fig. S3, ESI†), which means expanded interplanar spacing, hence benefitting the Na^+^ migration.^46,47^

In regard to the X-ray photoelectron spectroscopy (XPS) measurements, the obvious Mn signal at around 643.2 eV present in the survey spectra (Fig. 3c) of Mn-doped samples evidently verifies the Mn substitution in the framework. Fig. S4 (ESI†) illustrates the high-resolution Fe 2p, Ni 2p, and Mn 2p XPS spectra of Mn–NiHCF/ppy-2. Specifically, the characteristic peak of Fe 2p_1/2_ separates into Fe(ii) 2p_1/2_ (721.3 eV) and Fe(iii) 2p_1/2_ (723.5 eV), while the Fe 2p_3/2_ deconvolutes into Fe(ii) 2p_3/2_ (708.5 eV) and Fe(iii) 2p_3/2_ (710.1 eV), indicating the mixed states of Fe^2+^ and Fe^3+^.^24^ For the Ni 2p spectra, four main peaks can be obtained after deconvolution, including Ni(ii) 2p_1/2_ (874.1 eV), Ni(iii) 2p_1/2_ (876.2 eV), Ni(ii) 2p_3/2_ (856.4 eV), and Ni(iii) 2p_3/2_ (858.4 eV).^22^ In addition, the deconvolutions of Mn 2p_1/2_ at around 651.9 and 653.4 eV correspond to the Mn(ii) 2p_1/2_ and Mn(iii) 2p_1/2_, respectively, while those located at around 640.9 and 645.3 eV represent for Mn(ii) 2p_3/2_ and Mn(iii) 2p_3/2_.^48^ In principle, the Mn substitution in the framework, activating the electrochemical activity of NiHCF, provides abundant active sites and facilitates the faradaic redox reactions upon desalination.^49^

Fig. 3d depicts the Fourier transform infrared (FTIR) spectra of as-prepared samples. For the NiHCF, the characteristic infrared bands at 3640 cm^−1^ can be attributed to the stretching of –OH, while the bands at around 2163 and 2096 cm^−1^ correspond to the fingerprints of divalent iron (Fe(ii)) and trivalent iron (Fe(iii)), respectively, assigning to Fe^3+^–CN and Fe^2+^–CN.^7,11^ The band at 590 cm^−1^ represents the cyano group (–C≡N) stretching, whereas the metal–carbon bonding can be detected at around 455.0 cm^−1^.^33^ After Mn substitution and ppy connection, the stretching of –OH shows a slight right shift due to the enhanced interaction between –OH and –C≡N (–OH⋯N).^11^ Additionally, Mn substitution leads to the amalgamation of Fe^2+^–CN and Fe^3+^–CN into a single broad peak with a left shift, centered around 2066 cm^−1^.^50,51^ This observation is further supported by the FTIR spectra of pure MnHCF (Fig. S5, ESI†), where only one peak at approximately 2066 cm^−1^ is discernible, attributed to the stretching vibration of Fe^2+^–CN and Fe^3+^–CN, substantiating the rationale behind the merging of Fe^2+^–CN and Fe^3+^–CN bonds following Mn substitution.^35,50–52^ Moreover, the alterations in bonds configurations observed in the FTIR spectra provide additional confirmation of the substitution of Mn for Ni within the framework. Based on the above characterizations, the compositional and spatial control not only optimizes the inner structure of NiHCF but also induces improved interaction between the active framework and ppy substrate, thereby contributing to enhanced redox kinetics and high integrity of the hybrid electrode and enabling high desalination performance.^53,54^

Specifically, for the NiHCF and Mn–NiHCF, the ion/electron transfer in electrode largely depends on the contact of particles, that is, a particle-to-particle conductive pathway (Fig. S6a, ESI†).^55^ Nevertheless, the unsatisfied electronic conductivity of NiHCF and Mn–NiHCF not only restricts the charge transfer but also limits the utilization of redox sites, leading to poor desalination performance.^42^ Field emission scanning electron microscope (FESEM) observation for the NiHCF and Mn–NiHCF reveals that both of them show well-dispersed cube-shaped particulates with a size of about 150 nm (Fig. S6b–e†), which is in good agreement with earlier investigation,^24^ thus showing the successful synthesis of NiHCF and little influence of Mn substitution for the NiHCF framework.

**Fig. 4:**
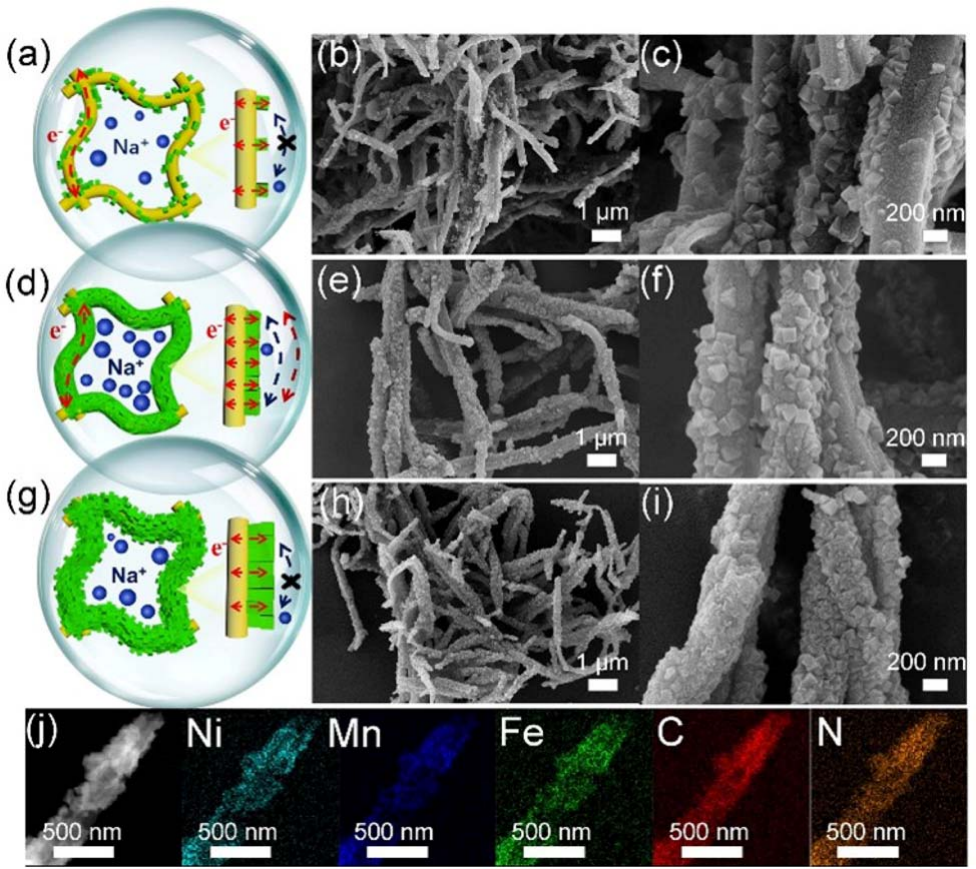
Illustration of the ionic/electronic conductivity and FESEM images with different magnifications of (a–c) Mn–NiHCF/ppy-1, (d–f) Mn–NiHCF/ppy-2, and (g–i) Mn–NiHCF/ppy-3; (j) elemental mapping of Mn–NiHCF/ppy-2.

After the introduction of ppy, the electronegativity of ppy induces the *in situ* nucleation of Mn–NiHCF on the surface of ppy,^56^ which can be clearly observed in Fig. 4. Compared with the pure NiHCF and Mn–NiHCF, the cubic particles on the ppy display similar size, and the number of detected particles gradually increases with the reduced addition of ppy in the precursor, showing high consistency with our design. Indeed, the tight hybridization between Mn–NiHCF and ppy can ensure high electronic conductivity.^23^ However, only short-range electronic/ionic conductivity at the contact area can be obtained for Fig. 4a–c, and the separated dispersion of Mn–NiHCF particles induces the lack of long-range conductivity, especially for ions.^42^ Furthermore, the substantial loading of ppy may potentially compromise the desalination capacity of the hybrid electrode, given that the primary contributor to desalination capacity is the NiHCF.^44^ Increasing the loading of Mn–NiHCF in the composite induces the homogeneous dispersion of Mn–NiHCF on the tubular ppy (Fig. 4d–f), simultaneously obtaining good contact between ppy and Mn–NiHCF particles, which leads to enhanced ionic/electronic conductivity.^32,46^ However, the excess of Mn–NiHCF causes aggregation of Mn–NiHCF particles (Fig. 4g–i).^21^ Noting that controlling the ppy loading changes the morphology of Mn–NiHCF hybrids, ensuring modified electronic/ionic conductivity and improved electrochemical performance.^22^ Concerning the elemental mapping, the uniform and co-existence of Ni, Mn, Fe, C, and N for the Mn–NiHCF/ppy-2 are obviously shown in Fig. 4j, which confirms the tight combination between Mn–NiHCF and ppy. As observed from Fig. S7 (ESI†), the electronic conductivities measured from the four-probe tests further reveal the increased electronic conductivity after Mn substitution and ppy connection, and the higher loading of ppy results in higher electronic conductivity. Given this, integrating conductive agents into PBAs serves the dual purpose of bolstering electronic conductivity while maintaining the structural integrity of the electrode, and this strategy is expected to notably elevate the electrochemical performance of the target electrode.

**Fig. 5:**
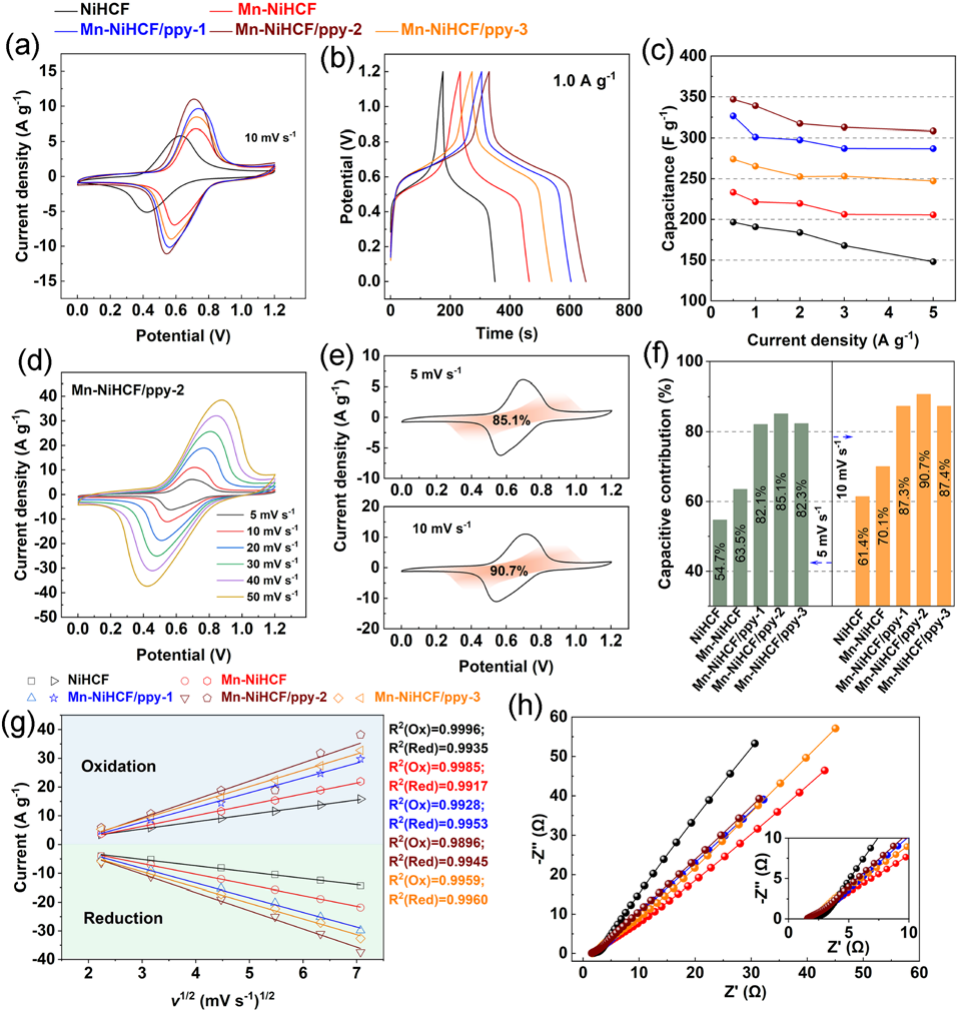
Electrochemical measurements of the NiHCF-based electrodes in 1.0 M Na_2_SO_4_ solution: (a) CV curves at 10 mV s^−1^; (b) galvanostatic discharge/charge profiles at the current density of 1.0 A g^−1^; (c) comparison of specific capacities at various current densities; CV curves of Mn–NiHCF/ppy-2 at (d) various scan rates and (e) capacitive contributions at 5.0 and 10.0 mV s^−1^; (f) comparison of capacitive contributions of the as-prepared five samples at 5.0 and 10.0 mV s^−1^; (g) fitting curves of peak current and square root of scan rate for all samples; (h) Nyquist plots for all samples with the inset of high-frequency zoom-in region.

To investigate the electrochemical properties of as-prepared samples, cyclic voltammetry (CV) and galvanostatic discharge/charge (GCD) measurements based on the three-electrode system in 1.0 M Na_2_SO_4_ solution have been performed. As shown in Fig. 5a, the redox reaction of high-spin Fe(iii)/Fe(ii) coordinated with N of cyano group (Fe–N≡C) upon Na^+^ insertion/desertion results in the redox pair at around 0.6 V, which can be clearly seen for all samples, thus revealing the reversible faradaic reactions of those NiHCF-based electrodes.^57^ The modifications in both composition and structure show different CVs compared with the pristine NiHCF electrode.^58^ Besides, the Mn–NiHCF/ppy-2 shows the maximum integrated area under the relevant CV curve, thus indicating the highest ion storage ability and thereby excellent desalination performance.^44^ Additionally, the higher ion storage ability of Mn–NiHCF/ppy-2 can be also demonstrated from the GCD profiles at 1.0 A g^−1^ (Fig. 5b) because the optimized Mn–NiHCF/ppy-2 displays the longest voltage plateau and charge/discharge time, further confirming its superiority in ion storage among all samples.^7^ Particularly, the specific capacitance decreases in the order of Mn–NiHCF/ppy-2 (338 F g^−1^) > Mn–NiHCF/ppy-3 (300 F g^−1^) > Mn–NiHCF/ppy-1 (265 F g^−1^) > Mn–NiHCF (221 F g^−1^) > NiHCF (190 F g^−1^) at 1.0 A g^−1^. Noting that the GCD profiles vary with different current densities, and the duration of charge/discharge plateau decreases at high current densities due to the fact that partial active sites cannot be utilized effectively (Fig. S8, ESI†).^6,11^ Moreover, the evident charge/discharge plateaus exhibiting high symmetry in the GCD profiles at various current densities for all samples, despite differences in duration, underscore the high electrochemical reversibility of the faradaic reaction within these electrodes.^18,20^Fig. 5c compares the specific capacitances of the as-fabricated five samples at various current densities, where the target Mn–NiHCF/ppy-2 exhibits superiority over those other samples on capacitances at all current densities (346, 338, 317, 312, and 308 F g^−1^ at 0.5, 1.0, 2.0, 3.0, 4.0, and 5.0 A g^−1^, respectively), showing excellent ion adsorption ability and potentially high desalination capacity.^56^

Generally, it is considered a typical capacitive-dominated electrochemical process (eqn (1)) when the specific current (*i*) in the CV curves shows a linear relationship with the scan rate (*v*).^6^ While the specific current varying with the square root of the scan rate (*v*^1/2^) represents the diffusion-controlled process.^22^

1 Capacitive-controlled electrochemical process: *i* = *k*_1_*v*

2 Diffusion-dominated electrochemical process: *i* = *k*_2_*v*^1/2^

where *k*_1_ and *k*_2_ are parameters varied by voltage, *i* corresponds to the specific current at a fixed voltage, and *v* represents the scan rate, separately.^59^ Specifically, both the diffusion and capacitive behaviors contribute to the current in CV except for the ideal battery and electrical double-layer capacitance materials, and the correlation between them can be explained as follows:

3 *i* = *k*_1_*v* + *k*_2_*v*^1/2^

which can be further modified into eqn (4) to facilitate the subsequent calculation.^59^

4 *i*/*v*^1/2^ = *k*_1_*v*^1/2^ + *k*_2_

Based on the above discussion, after solving for *k*_1_ and *k*_2_ from the slope and the *Y*-axis intercept by fitting a straight line across multiple scan rates at each specific potential, the contributions for the current induced by capacitive or diffusion behaviors can be determined, hence allowing to separate the CV into the capacitive-controlled and diffusion-dominated regions.^60^ In principle, the surface-related kinetics behaviors correspond to the capacitive process, while the solid-state diffusion process often accompanies with slow mass transfer.^61^ Based on the CV curves of Mn–NiHCF/ppy-2 electrode at various scan rates (Fig. 5d), the capacitive contributions at 5.0 and 10.0 mV s^−1^ are 85.1% and 90.7%, respectively, after data collection and fitting (Fig. 5e). Besides, the comparison of capacitive and diffusion contribution of all prepared NiHCF electrodes at 5 and 10 mV s^−1^ according to the CV curves in Fig. S9 (ESI†) has been illustrated in Fig. 5f and S10† (ESI†), where the capacitive contribution follows the order: Mn–NiHCF/ppy-2 > Mn–NiHCF/ppy-3 > Mn–NiHCF/ppy-1 > Mn–NiHCF > NiHCF. It's important to highlight that the clustered Mn–NiHCF on the ppy surface in Mn–NiHCF/ppy-3 diminishes the exposed surface area, limiting ionic/electronic migration. Consequently, this results in a lower capacitive contribution compared to Mn–NiHCF/ppy-2.^62^ The substantial capacitive contribution observed in the charge storage of Mn–NiHCF/ppy-2 underscores the efficacy of the rational design involving Mn substitution and ppy connection. This design proves advantageous in achieving rapid redox kinetics and a high ion storage capacity, thereby facilitating the subsequent desalination process.^11^

By means of CV curves at various scan rates, the investigation of Na^+^ transportation process on the surface of various electrodes was also performed. The higher peak currents after Mn substitution and ppy connection at the same scan rate indicate the improved charge transfer and increased electrochemical activity.^63,64^ Regarding the linear fitting results of peak current *versus* square root of scan rate (Fig. 5g), the high index (*R*^2^ > 0.99) for the fitting reveals that all the five samples undergo the same ionic diffusion mechanism, all of which is controlled by surface diffusion.^65^ Specifically, the exact ionic diffusion coefficient can be obtained based on the following equation:5
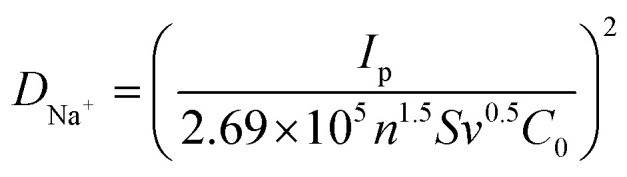
where, *I*_p_ (A), *n*, *S* (cm^2^), *v* (mV s^−1^), and *C*_0_ (mol L^−1^) represent the peak current, the number of electrons involved in the redox reaction, the load area of active material, the scan rate, and the concentration of Na^+^.^66^ Table S1 (ESI†) compares the Na^+^ diffusion coefficient of as-prepared five samples, where the Mn substituted sample (Mn–NiHCF) exhibits a larger average coefficient (1.73 × 10^−10^ cm^2^ s^−1^) compared with pristine NiHCF (7.93 × 10^−11^ cm^2^ s^−1^). Additionally, the introduction of ppy induces the further increase of diffusion coefficient, and the desired Mn–NiHCF/ppy-2 exhibits the highest diffusion coefficient of 5.76 × 10^−10^ cm^2^ s^−1^.^66^ Additionally, the Nyquist plots of all as-prepared samples (Fig. 5h) reveal that the target Mn–NiHCF/ppy-2 possesses the lowest charge transfer resistance (*R*_ct_) based on the smallest semi-circle in the high-frequency region, demonstrating superior charge transfer of Mn–NiHCF/ppy-2, which shows high agreement with the calculation and characterization results.^67^

*In situ* EIS measurements were further employed to estimate the detailed evolution of charge transfer kinetics, taking the optimized Mn–NiHCF/ppy-2 as an example. For the charging process, as shown in Fig. S11a (ESI†), the quasi-semicircle in the high-frequency region, corresponding to the *R*_ct_, is restricted, while the obvious slope line in the low-frequency region indicates favorable ionic diffusion on the surface of the electrode at the initial charge, demonstrating ion storage by EDL in this stage.^3,60^ After charging to 0.6 V, the obvious difference can be detected from the appearance of the quasi-semicircle and the restriction of the slope line, which manifests that the charge transfer resistance dominates the faradaic reactions.^65^ Further charging to 1.2 V, away from the redox region, results in the vanish of quasi-semicircle and convenient ionic diffusion. Similar to the charging process, the *in situ* EIS measurement for the discharge process (Fig. S11b, ESI†) also displays the capacitive feature at the region away from the redox reaction, and the charge transfer controls the faradaic reaction.^68^ In summary, the ion storage by synergistic effect of faradaic reaction and EDL can not only guarantee high capacitance but also induce fast redox kinetics, hence contributing to modifying the desalination performance.^6^

**Fig. 6:**
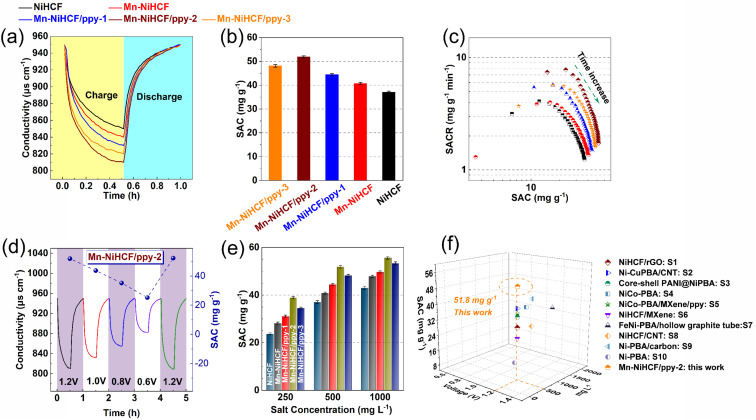
(a) Conductivity–time plots of the first desalination/regeneration with various NiHCF-based electrodes in 500 mg per L NaCl solution at 1.2 V and (b) corresponding salt adsorption capacities; (c) desalination Ragone plots of as-prepared five NiHCF-based electrodes; (d) conductivity *versus* time profiles and SACs of the Mn–NiHCF/ppy-2 electrode in 500 mg per L NaCl solution with various voltages in CDI cell; (e) SACs of NiHCF-based electrodes *versus* salt concentration; (f) comparison of desalination performance between the target Mn–NiHCF/ppy-2 and other MHCF-based electrodes in CDI.

In general, the batch mode was employed for all desalination experiments, utilizing the active carbon and NiHCF-based composites as the positive and negative electrodes, respectively. A feedwater NaCl concentration of 500 mg L^−1^ and a voltage of 1.2 V were applied for the CDI cell to initially evaluate the desalination performance of the fabricated samples. As depicted, two typical stages, which involve electrochemical adsorption followed by desorption over one charge/discharge cycle (*i.e.*, one desalination–regeneration cycle), could be observed from the conductivity–time profiles for all NiHCF electrodes (Fig. 6a). The Mn–NiHCF/ppy-2 electrode exhibites a much faster and higher Na^+^ ion capture ability compared to other electrodes during the desalination process. Besides, further comparison on the salt adsorption capacity (SAC) after quantitative calculation (Fig. 6b) demonstrates that the NiHCF/ppy-2 delivers the highest SAC of 51.8 mg g^−1^, showing superior to other electrodes (37.0 mg g^−1^ for NiHCF, 40.7 mg g^−1^ for Mn–NiHCF, 44.4 mg g^−1^ for Mn–NiHCF/ppy-1, and 48.1 mg g^−1^ for Mn–NiHCF/ppy-3). In principle, the Mn substitution promotes both the ionic diffusion and electronic conductivity in the framework, combining with the optimized loading of ppy to guarantee the stability and integrity of the electrode, contributing to the high Na^+^ capture.^44^ Moreover, the *in situ* growth of Mn–NiHCF on tubular ppy not only enhances the interfacial charge transfer but also improves the interaction between them, thus simultaneously obtaining modified redox kinetics and tight combination.^7,59^ Noting that the SACs of Mn–NiHCF/ppy electrodes are found to have a significant dependence on ppy loading since insufficient ppy connection could not ensure good electronic conductivity or provide enough buffering matrices, while excessive ppy loading would sacrifice the high electrochemical activity of NiHCF.^7^

Fig. 6c compares the salt adsorption rate (SAR) and SAC based on CDI Ragone plots (SAR *versus* SAC) among the prepared samples, from which we can get the conclusion that superior desalination performance can be obtained when the SAR *versus* SAC profile gets closer to the upper right corner in the Ragone plots.^68^ Noting that the distance of these SAR *versus* SAC profiles follow the order of NiHCF < Mn–NiHCF < Mn–NiHCF/ppy-1 < Mn–NiHCF/ppy-3 < Mn–NiHCF/ppy-2, thus manifesting that the desired Mn–NiHCF/ppy-2 possesses both the fastest and highest Na^+^ capture ability, further highlighting the enhanced redox kinetics and modified electrochemical activity for NiHCF. Fig. S12 (ESI†) depicts the current response upon desalination of various NiHCF-based electrodes at 1.2 V in 500 mg L^−1^ feedwater, based on which the charge efficiencies are calculated to be 0.50, 0.60, 0.68, 0.81, and 0.75, respectively, for NiHCF, Mn–NiHCF, Mn–NiHCF/ppy-1, Mn–NiHCF/ppy-2, and Mn–NiHCF/ppy-3. Overall, the modified redox kinetics and improved integrity of the electrode endow the target Mn–NiHCF/ppy-2 with improved charge transfer and enhanced desalination performance.^69^

To reveal the potential for practical application of the as-fabricated CDI cell, the desalination performance under various voltages and feed concentrations was evaluated. Fig. 6d and S13 (ESI†) show that the conductivities and corresponding SACs of all NiHCF-based electrodes are strongly depended on the voltage employed from 0.6 to 1.2 V. For all samples, the decline in conductivity indicates the capture of Na^+^, while the various modifications applied to the NiHCF show differing in SACs. Specifically, for the optimized Mn–NiHCF/ppy-2, the SAC decreases from 52.2 to 25.2 mg g^−1^ when the voltage drops from 1.2 to 0.6 V (Table S2, ESI†). It is worth noting that when the voltage is returned to 1.2 V, the SAC of Mn–NiHCF/ppy-2 can be recovered to 51.8 mg g^−1^. In principle, the higher voltage employed for the CDI cell can improve performance due to enhanced sorption ability.^70^ However, side reactions also occur, degrading the cycling performance and reducing the charge efficiency.^7^ Based on the literature review of Na^+^ capturing electrodes for CDI, the cell voltage of 1.2 V was often selected to estimate the desalination performance, and therefore was adopted as the optimized voltage to investigate the effect of feedwater salt concentration for SAC (Fig. 6e and S14 in ESI†).^60^ With the increase of salt concentration, the electrodes manifest rapid adsorption/desorption equilibrium as well as improved SAC. Noting that the highest salt concentration of 1000 mg L^−1^ results in the highest SAC, the results from which show high consistency with the previously related studies.^21–23^ Additionally, the target Mn–NiHCF/ppy-2, featuring modified redox kinetics and good electrode integrity, exhibits the highest SAC among all NiHCF-based electrodes, showing desalination capacity of 38.8, 51.8, and 55.5 mg g^−1^ with the feedwater salt concentration of 250, 500, and 1000 mg g^−1^, respectively (Table S3, ESI†). Compared with other MHCF electrodes reported previously, the optimized Mn–NiHCF/ppy-2 demonstrates superiority on SAC at 1.2 V with the feedwater salt concentration of 500 mg L^−1^ (Fig. 6f), thus indicating the significance of compositional and structural control to modify the desalination performance of NiHCF.^57^

**Fig. 7:**
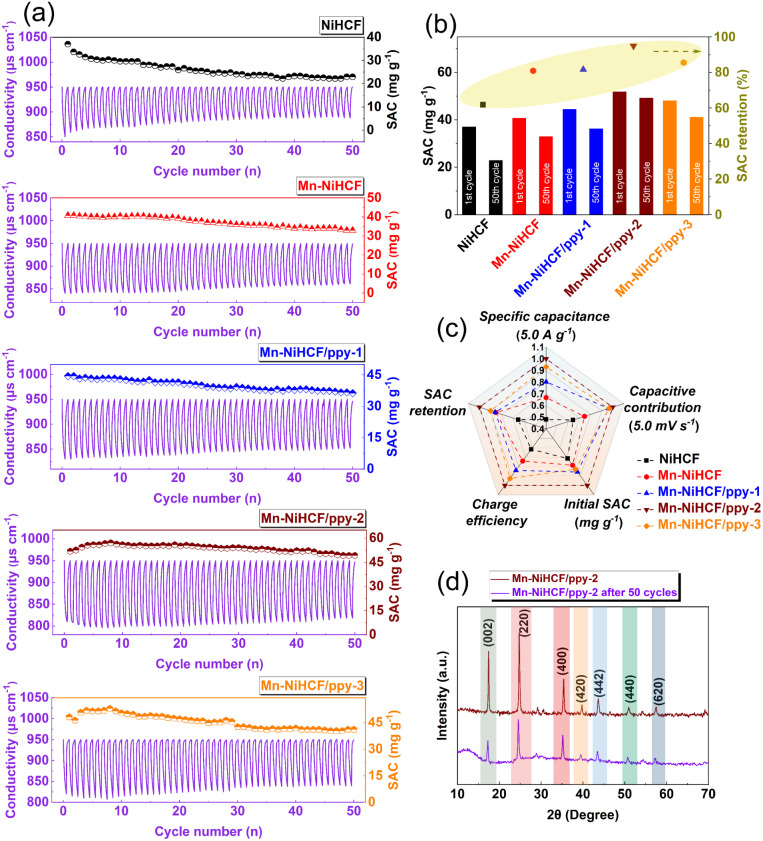
(a) Cycling performance of all NiHCF-based electrodes for 50 adsorption/desorption cycles in 500 mg per L NaCl solution; (b) comparison of initial/final SACs and corresponding SAC retention for the as-prepared NiHCF-based electrodes; (c) systematic comparison on specific capacitance, SAC retention, charge efficiency, initial SAC, and capacitive contribution of as-prepared NiHCF electrodes (the data has been normalized); (d) comparison of XRD pattern of the Mn–NiHCF/ppy-2 before cycling and after 50 cycles.

Except for the SAC, another index to evaluate the CDI performance is cycling stability, which plays an important role in practical application of CDI. In this regard, the cycling performances of CDI cells with various NiHCF-based electrodes have been observed through repeated charge and discharge in 500 mL g^−1^ salt concentration at 1.2 V. Fig. 7a shows the feedwater conductivity and corresponding SAC plotted as a function of cycling number for all NiHCF-based electrodes. For the pristine NiHCF electrode, the SAC reduces from 37.0 to 22.9 mg g^−1^ from the initial cycle to the 50th cycle, with a capacity retention of 61.8% (Fig. 7b), showing degraded cycling stability. Interestingly, the Mn substitution (Mn–NiHCF) modifies the cycling stability, with a desalination capacity of 32.9 mg g^−1^ after 50 cycles and 80.8% capacity retention. Further combined with ppy, the high conductivity and efficient buffering induce enhanced desalination capacity and capacity retention, and the target Mn–NiHCF/ppy-2 shows the highest SAC of 49.2 mg g^−1^ after 50 cycles and 94.9% capacity retention, further highlighting the significance of composition/structure modification for desalination performance. Noting that the excess loading of NiHCF (Mn–NiHCF/ppy-3) displays inferiority on both SAC and retention compared with Mn–NiHCF/ppy-2, which can be ascribed to the fact that a large amount of NiHCF on ppy may restrict the migration of Na^+^, resulting in increased electrochemical polarization.^69^

To highlight the advantages of Mn–NiHCF/ppy-2 for other NiHCF-based electrodes in this work, a detailed comparison of electrochemical and desalination performance (Fig. 7c) demonstrates that the Mn–NiHCF/ppy-2 shows the superiorities on specific capacitance, capacitive contribution, SAC, SAC retention after 50 cycles, and charge efficiency over the other NiHCF electrodes. To confirm the enhanced structure stability of the NiHCF electrode after Mn substitution and ppy connection, the characterizations on XRD and FTIR before and after cycling for the Mn–NiHCF/ppy-2 have been conducted. As shown in Fig. 7d, the crystal structure of NiHCF is well maintained after 50 cycles since no new phases can be detected, while the results of FTIR reveal that the main functional groups of the pristine electrode can be well-maintained after 50 cycles (Fig. S15, ESI†), thus showing the excellent structure stability of Mn–NiHCF/ppy-2 and its high promise for efficient desalination. Overall, the outstanding desalination performance exhibited by Mn–NiHCF/ppy-2 underscores the synergistic effect of Mn substitution and ppy connection, and such modifications represent typical compositional and morphological tailoring approaches, showcasing their feasibility and promise in enhancing the desalination performance of the PBAs electrode.

## Conclusion

Herein, we strategically prepare a new hybrid structure of Mn–NiHCF/ppy. From both theoretical calculation and experimental measurement, we have demonstrated modified dynamics for both ions and electrons, improved electrochemical activity, and enhanced desalination performance. Particularly, the introduction of partial Mn into the NiHCF reduces both the diffusion energy of Na^+^ in the cubic framework and the bandgap between conduction and valence bands, while the ppy substrate connects the separated NiHCF, further guaranteeing the conductivity and integrity of the hybrid electrode. Our concept leads to enhanced electrochemical activity and desalination performance. Importantly, the tailoring strategies reported in this work are also feasible for other faradaic electrodes for efficient desalination, hence promoting the development and practical application of CDI.

## Data availability

The data that support the findings of this study are available in the main text and ESI.†

## Author contributions

Jiabao Li: investigation, methodology, data curation, writing – original draft; Ruoxing Wang: validation, investigation; Lanlan Han: project administration; Tianyi Wang: methodology; Zeinhom M. El-Bahy: writing – review & editing; Yiyong Mai: writing – review & editing; Chengyin Wang: data curation, formal analysis; Yusuke Yamauchi: conceptualization, validation; Xingtao Xu: supervision, conceptualization, writing-review & editing.

## Conflicts of interest

There are no conflicts to declare.

## Supplementary Material

SC-015-D4SC00686K-s001

## References

[cit1] He C., Liu Z., Wu J., Pan X., Fang Z., Li J., Bryan B. A. (2021). Future global urban water scarcity and potential solutions. Nat. Commun..

[cit2] Srimuk P., Su X., Yoon J., Aurbach D., Presser V. (2020). Charge-transfer materials for electrochemical water desalination, ion separation and the recovery of elements. Nat. Rev. Mater..

[cit3] Gamaethiralalage J. G., Singh K., Sahin S., Yoon J., Elimelech M., Suss M. E., Liang P., Biesheuvel P. M., Zornitta R. L., de Smet L. C. P. M. (2021). Recent advances in ion selectivity with capacitive deionization. Energy Environ. Sci..

[cit4] Xu X., Eguchi M., Asakura Y., Pan L., Yamauchi Y. (2023). Metal-organic framework derivatives for promoted capacitive deionization of oxygenated saline water. Energy Environ. Sci..

[cit5] Liu X., Xu X., Xuan X., Xia W., Feng G., Zhang S., Wu Z. G., Zhong B., Guo X., Xie K., Yamauchi Y. (2023). Unlocking Enhanced Capacitive Deionization of NaTi_2_(PO_4_)_3_/Carbon Materials by the Yolk-Shell Design. J. Am. Chem. Soc..

[cit6] Du J., Xing W., Yu J., Feng J., Tang L., Tang W. (2023). Synergistic effect of intercalation and EDLC electrosorption of 2D/3D interconnected architectures to boost capacitive deionization for water desalination *via* MoSe_2_/mesoporous carbon hollow spheres. Water Res..

[cit7] Meng F., Ding Z., Xu X., Liu Y., Lu T., Pan L. (2023). Metal organic framework-derived nitrogen-doped porous carbon sustained Prussian blue analogues for efficient and fast hybrid capacitive deionization. Sep. Purif. Technol..

[cit8] Zhang X., Li Y., Yang Z., Yang P., Wang J., Shi M., Yu F., Ma J. (2022). Industrially-prepared carbon aerogel for excellent fluoride removal by membrane capacitive deionization from brackish groundwaters. Sep. Purif. Technol..

[cit9] Liu Y., Wang K., Xu X., Eid K., Abdullah A. M., Pan L., Yamauchi Y. (2021). Recent Advances in Faradic Electrochemical Deionization: System Architectures *versus* Electrode Materials. ACS Nano.

[cit10] Gong S., Liu H., Zhao F., Zhang Y., Xu H., Li M., Qi J., Wang H., Li C., Peng W., Fan X., Liu J. (2023). Vertically Aligned Bismuthene Nanosheets on MXene for High-Performance Capacitive Deionization. ACS Nano.

[cit11] Bao Y., Hao J., Zhang S., Zhu D., Li F. (2023). Structural/Compositional-Tailoring of Nickel Hexacyanoferrate Electrodes for Highly Efficient Capacitive Deionization. Small.

[cit12] Wang Q., Wu Q., Zhao M., Lu S., Liang D. (2024). Prussian blue analogue based integrated membrane electrodes for desalination and selective removal of ammonium ions in a rocking-chair capacitive deionization. Chem. Eng. J..

[cit13] Gao Y., Li Z., Fu Z., Zhang H., Wang G., Zhou H. (2021). Highly selective capacitive deionization of copper ions in FeS_2_@N, S co-doped carbon electrode from wastewater. Sep. Purif. Technol..

[cit14] Zheng S.-M., Li B., Yuan Z.-H., Yang J.-C. E., Zhang J., Zhong L.-B., Zheng Y.-M. (2023). Zinc oxide nanosheet decorated self-supporting hierarchical porous wood carbon electrode for efficient capacitive deionization defluorination. Sep. Purif. Technol..

[cit15] Yang Z., Yang P., Zhang X., Yin H., Yu F., Ma J. (2023). Two-Dimensional Hetero-structured TiO_2_/TiS_2_ Nanosheets for Capacitive Deionization. Chem. Mater..

[cit16] Tang Z., Hu B., Nie P., Shang X., Yang J., Liu J. (2023). Bimetallic Fe, Ni-PBA on hollow graphite tube for capacitive deionization with exceptional stability. Chem. Eng. J..

[cit17] Guo J., Xu X., Hill J. P., Wang L., Dang J., Kang Y., Li Y., Guan W., Yamauchi Y. (2021). Graphene-carbon 2D heterostructures with hierarchically-porous P, N-doped layered architecture for capacitive deionization. Chem. Sci..

[cit18] Shi W., Liu X., Deng T., Huang S., Ding M., Miao X., Zhu C., Zhu Y., Liu W., Wu F., Gao C., Yang S. W., Yang H. Y., Shen J., Cao X. (2020). Enabling Superior Sodium Capture for Efficient Water Desalination by a Tubular Polyaniline Decorated with Prussian Blue Nanocrystals. Adv. Mater..

[cit19] Tu X., Liu Y., Wang K., Ding Z., Xu X., Lu T., Pan L. (2023). Ternary-metal Prussian blue analogues as high-quality sodium ion capturing electrodes for rocking-chair capacitive deionization. J. Colloid Interface Sci..

[cit20] Wang S., Li Z., Wang G., Wang Y., Ling Z., Li C. (2022). Freestanding Ti_3_C_2_T_x_ MXene/Prussian Blue Analogues Films with Superior Ion Uptake for Efficient Capacitive Deionization by a Dual Pseudocapacitance Effect. ACS Nano.

[cit21] Zhang X., Dutta J. (2021). X-Fe (X = Mn, Co, Cu) Prussian Blue Analogue-Modified Carbon Cloth Electrodes for Capacitive Deionization. ACS Appl. Energy Mater..

[cit22] Xu L., Ding Z., Chen Y., Xu X., Liu Y., Li J., Lu T., Pan L. (2023). Carbon nanotube bridged nickel hexacyanoferrate architecture for high-performance hybrid capacitive deionization. J. Colloid Interface Sci..

[cit23] Chen Z., Ding Z., Chen Y., Xu X., Liu Y., Lu T., Pan L. (2023). Three-dimensional charge transfer pathway in close-packed nickel hexacyanoferrate-on-MXene nano-stacking for high-performance capacitive deionization. Chem. Eng. J..

[cit24] Guo J., Wang Y., Zhang H., Cai Y., Fang R. (2023). Hollow core-shell PANI-encapsuled Ni-Prussian blue analogue (H-NP@PANI) with omnidirectional conductive layer for efficient capacitive desalination. Desalination.

[cit25] Wang S., Wang G., Wang Y., Song H., Lv S., Li T., Li C. (2020). *In Situ* Formation of Prussian Blue Analogue Nanoparticles Decorated with Three-Dimensional Carbon Nanosheet Networks for Superior Hybrid Capacitive Deionization Performance. ACS Appl. Mater. Interfaces.

[cit26] braWei X., Zhao Y., Liang B., Mo X., Li K. (2021). Core-shell nanoparticles of Prussian blue analogues as efficient capacitive deionization electrodes for brackish water desalination. Sep. Purif. Technol..

[cit27] Ding Z., Xu X., Li Y., Wang K., Lu T., Pan L. (2019). Significantly improved stability of hybrid capacitive deionization using nickel hexacyanoferrate/reduced graphene oxide cathode at low voltage operation. Desalination.

[cit28] Eguchi M., Han M., Asakura Y., Hill J. P., Henzie J., Ariga K., Rowan A. E., Chaikittisilp W., Yamauchi Y. (2023). Materials Space-Tectonics: Atomic-level Compositional and Spatial Control Methodologies for Synthesis of Future Materials. Angew. Chem., Int. Ed..

[cit29] Liu J., Liu J., Tang M., Fu J., Kuang X., Ma J. (2024). Boosting Sodium Storage in Prussian Blue Analogs Through Iron Vacancies and Copper Doping. Adv. Funct. Mater..

[cit30] Zheng Y., Zhang R., Zhang L., Gu Q., Qiao Z. A. (2021). A Resol-Assisted Cationic Coordinative Co-assembly Approach to Mesoporous ABO_3_ Perovskite Oxides with Rich Oxygen Vacancy for Enhanced Hydrogenation of Furfural to Furfuryl Alcohol. Angew. Chem., Int. Ed..

[cit31] Xue J., Zhang Z., Li H. (2024). The enhanced synergistic capacitive desalination enabled by Ni@NiO@C hybrid anode. Sep. Purif. Technol..

[cit32] Zhang L. L., Wei C., Fu X. Y., Chen Z. Y., Yan B., Sun P. P., Chang K. J., Yang X. L. (2021). Ternary Ni-based Prussian blue analogue with superior sodium storage performance induced by synergistic effect of Co and Fe. Carbon Energy.

[cit33] Xu Z., Sun Y., Xie J., Nie Y., Xu X., Tu J., Zhang J., Qiu L., Zhu T., Zhao X. (2022). Scalable Preparation of Mn/Ni Binary Prussian Blue as Sustainable Cathode for Harsh-Condition-Tolerant Sodium-Ion Batteries. ACS Sustainable Chem. Eng..

[cit34] Du M., Geng P., Pei C., Jiang X., Shan Y., Hu W., Ni L., Pang H. (2022). High-Entropy Prussian Blue Analogues and Their Oxide Family as Sulfur Hosts for Lithium-Sulfur Batteries. Angew. Chem., Int. Ed..

[cit35] Feng S., Wang J., Gao N., Wen J., Li X., Xiao J. (2022). Heterogeneous interface of Ni-Mn composite Prussian blue analog-coated structure modulates the adsorption and conversion of polysulfides in lithium-sulfur batteries. Electrochim. Acta.

[cit36] Jiang W., Wang T., Chen H., Suo X., Liang J., Zhu W., Li H., Dai S. (2021). Room temperature synthesis of high-entropy Prussian blue analogues. Nano Energy.

[cit37] Zhu Y., Xu F., Sun L., Lao J., Shao Q., Luo Y., Fang S., Chen Y., Yu C., Chu H., Pan H., Cao Z., Zeng J. (2024). 3D nanocubes NiCo-PBA sulfide for high-performance supercapacitors. Electrochim. Acta.

[cit38] Oh G., Kim J., Kansara S., Kang H., Jung H.-G., Sun Y.-K., Hwang J.-Y. (2024). Experimental and computational optimization of Prussian blue analogues as high-performance cathodes for sodium-ion batteries: a review. J. Energy Chem..

[cit39] Liu J., Shen Z., Lu C.-Z. (2024). Research progress of Prussian blue and its analogues for cathodes of aqueous zinc ion batteries. J. Mater. Chem. A.

[cit40] Shu W., Han C., Wang X. (2023). Prussian Blue Analogues Cathodes for Nonaqueous Potassium-Ion Batteries: Past, Present, and Future. Adv. Funct. Mater..

[cit41] Zeng Y., Lu X., Zhang S., Luan D., Li S., Lou X. (2021). Construction of Co-Mn Prussian Blue Analog Hollow Spheres for Efficient Aqueous Zn-ion Batteries. Angew. Chem., Int. Ed..

[cit42] Xu X., Tang J., Qian H., Hou S., Bando Y., Hossain M. S. A., Pan L., Yamauchi Y. (2017). Three-Dimensional Networked Metal-Organic Frameworks with Conductive Polypyrrole Tubes for Flexible Supercapacitors. ACS Appl. Mater. Interfaces.

[cit43] Chu M., Wang Y., Xin J., Chu D., Liu Y., Song D., Yang G., Ma H., Pang H., Wang X. (2024). Preparation of a three-dimensional bimetallic CoFe_x_/N-doped carbon nanomaterial used as modified electrode for the efficient electrochemical detection of l-tyrosine. Mater. Chem. Phys..

[cit44] Meng F., Tu X., Liu Y., Wang K., Xu X., Liu X., Gong Z., Lu T., Pan L. (2024). Carbon nanotube sustained ternary-metal Prussian blue analogues for superior-performance rocking-chair capacitive deionization. Sep. Purif. Technol..

[cit45] Shang J., Ma C., Zhang C., Zhang W., Shen B., Wang F., Guo S., Yao S. (2024). Separator Modified by Carbon-Encapsulated CoFe Alloy Nanoparticles Supported on Carbon Nanotubes for Advanced Lithium-Sulfur Batteries. ACS Appl. Nano Mater..

[cit46] Zhang L.-L., Chen Z.-Y., Fu X.-Y., Yan B., Tao H.-C., Yang X.-L. (2022). Effect of Zn-substitution induced structural regulation on sodium storage performance of Fe-based Prussian blue. Chem. Eng. J..

[cit47] Liu R., Wang Y., Wu Y., Ye X., Cai W. (2023). Controllable synthesis of nickel–cobalt-doped Prussian blue analogs for capacitive desalination. Electrochim. Acta.

[cit48] Zhang X., Toledo-Carrillo E. A., Yu D., Dutta J. (2022). Effect of Surface Charge on the Fabrication of Hierarchical Mn-Based Prussian Blue Analogue for Capacitive Desalination. ACS Appl. Mater. Interfaces.

[cit49] Wang Y., Jiang N., Yang C., Liu J., Sun S., Wang X., Yang J., Liu Y. (2024). High-entropy Prussian blue analogs with 3D confinement effect for long-life sodium-ion batteries. J. Mater. Chem. A.

[cit50] Li A., Duan L., Liao J., Sun J., Man Y., Zhou X. (2022). Formation of Mn–Ni Prussian Blue Analogue Spheres as a Superior Cathode Material for Potassium-Ion Batteries. ACS Appl. Energy Mater..

[cit51] Xiong P., Zeng G., Zeng L., Wei M. (2015). Prussian blue analogues Mn[Fe(CN)_6_]_0.6667_·*n*H_2_O cubes as an anode material for lithium-ion batteries. Dalton Trans..

[cit52] Singh K., Li G., Lee J., Zuilhof H., Mehdi B. L., Zornitta R. L., de Smet L. C. P. M. (2021). Divalent Ion Selectivity in Capacitive Deionization with Vanadium Hexacyanoferrate: Experiments and Quantum-Chemical Computations. Adv. Funct. Mater..

[cit53] Khoi T. M., Kim J., Tran N. A. T., Huynh V. P., Lee Y.-W., Cho Y. (2024). Redox flow deionization using Prussian blue and functionalized ion exchange membrane for enhanced selective ion recovery. Desalination.

[cit54] Wu Y., Huang J., Li C., Wang W. (2024). Structural distortion-induced monoclinic sodium iron hexacyanoferrate as a high-performance electrode for rocking-chair desalination batteries. Nanoscale.

[cit55] Xing Z., Xuan X., Hu H., Li M., Gao H., Alowasheeir A., Jiang D., Zhu L., Li Z., Kang Y., Zhang J., Yi X., Yamauchi Y., Xu X. (2023). Particle size optimization of metal-organic frameworks for superior capacitive deionization in oxygenated saline
water. Chem. Commun..

[cit56] Cai Y., Zhang W., Zhao J., Wang Y. (2023). Flexible structural construction of the ternary composite Ni, Co-Prussian blue analogue@MXene/polypyrrole for high-capacity capacitive deionization. Appl. Surf. Sci..

[cit57] Zhao Y., Liang B., Wei X., Li K., Lv C., Zhao Y. (2019). A core–shell heterostructured CuFe@NiFe Prussian blue analogue as a novel electrode material for high-capacity and stable capacitive deionization. J. Mater. Chem. A.

[cit58] Ren Y., Yu F., Li X. G., Yuliarto B., Xu X., Yamauchi Y., Ma J. (2023). Soft-hard interface design in super-elastic conductive polymer hydrogel containing Prussian blue analogues to enable highly efficient electrochemical deionization. Mater. Horiz..

[cit59] Li J., Tang S., Li Z., Wang C., Li J., Li X., Ding Z., Pan L. (2021). Crosslinking Nanoarchitectonics of Nitrogen-doped Carbon/MoS_2_ Nanosheets/Ti_3_C_2_T_x_ MXene Hybrids for Highly Reversible Sodium Storage. ChemSusChem.

[cit60] Li J., Ding Z., Pan L., Li J., Wang C., Wang G. (2021). Facile self-templating synthesis of layered carbon with N, S dual doping for highly efficient sodium storage. Carbon.

[cit61] Li J., Tang S., Li Z., Wang C., Pan L. (2021). Boosting the lithium storage performance by synergistically coupling ultrafine heazlewoodite nanoparticle with N, S co-doped carbon. J. Colloid Interface Sci..

[cit62] Chen Z., Xu X., Liu Y., Li J., Wang K., Ding Z., Meng F., Lu T., Pan L. (2022). Ultra-durable and highly-efficient hybrid capacitive deionization by MXene confined MoS_2_ heterostructure. Desalination.

[cit63] Quan J., Xu E., Zhu H., Chang Y., Zhu Y., Li P., Sun Z., Yu D., Jiang Y. (2021). A Ni-doping-induced phase transition and electron evolution in cobalt hexacyanoferrate as a stable cathode for sodium-ion batteries. Phys. Chem. Chem. Phys..

[cit64] Cao J., Wang Y., Wang L., Yu F., Ma J. (2019). Na_3_V_2_(PO_4_)_3_@C as Faradaic Electrodes in Capacitive Deionization for High-Performance Desalination. Nano Lett..

[cit65] Gu J., Chen L., Li X., Luo G., Fan L., Chao Y., Ji H., Zhu W. (2024). Multifunctional AlPO_4_ reconstructed LiMn_2_O_4_ surface for electrochemical lithium extraction from brine. J. Energy Chem..

[cit66] Luo G., Li X., Chen L., Zhang Y., Gu J., Chao Y., Zhu W., Liu Z., Xu C. (2023). Island-like CeO_2_ decorated LiMn_2_O_4_: surface modification enhancing electrochemical lithium extraction and cycle performance. Chem. Eng. J..

[cit67] Zhang Y., Wu J., Zhang S., Shang N., Zhao X., Alshehri S. M., Ahamad T., Yamauchi Y., Xu X., Bando Y. (2022). MOF-on-MOF nanoarchitectures for selectively functionalized nitrogen-doped carbon-graphitic carbon/carbon nanotubes heterostructure with high capacitive deionization performance. Nano Energy.

[cit68] Xu L., Liu Y., Ding Z., Xu X., Liu X., Gong Z., Li J., Lu T., Pan L. (2024). Solvent-Free Synthesis of Covalent Organic Framework/Graphene Nanohybrids: High-Performance Faradaic Cathodes for Supercapacitors and Hybrid Capacitive Deionization. Small.

[cit69] Shen X., Xiong Y., Yu F., Ma J. (2023). Chinese dumpling-like NaTi_2_(PO_4_)_3_/MXene@reduced graphene oxide for capacitive deionization with high capacity and good cycling stability. J. Mater. Chem. A.

[cit70] Huo S., Zhang P., He M., Zhang W., Liang B., Zhang M., Wang H., Li K. (2021). Sustainable development of ultrathin porous carbon nanosheets with highly accessible defects from biomass waste for high-performance capacitive desalination. Green Chem..

