# Supplementary material for: Enhanced redox kinetics of Prussian blue analogues for superior electrochemical deionization performance
Source: Chem Sci. 2024 Jun 4;15(30):11814–24. doi: 10.1039/d4sc00686k (PMC11290438; doi:10.1039/d4sc00686k)
Supplement: SC-015-D4SC00686K-s001 [file SC-015-D4SC00686K-s001.pdf]

Electronic Supplementary Material (ESI) for Chemical Science.
This journal is © The Royal Society of Chemistry 2024

Experimental section

Preparation of ppy tube: with modifications, the ppy tubes were synthesized according to a previous report.[1] First, 0.05 g of MO was dispersed into 50 mL of deionized water, followed by the addition of 0.24 g of $FeCl_3 \cdot 6H_2O$ under stirring in an ice bath. Then, 0.1 mL of pyrrole was injected into the mixture, which was continuously stirred for 24 h in the dark. Afterwards, the product was collected and washed with deionized water and ethanol before being dried at 60 °C overnight.

Material Characterization

The crystallographic structures of as-prepared samples were checked by the X-ray diffraction (XRD, XRD-6100 diffractometer, Shimadzu, Japan) measurements with the tube voltage and current of 40 kV and 30 mA, respectively. The Fourier transform infrared (FTIR) spectra were collected from the infrared spectrometer (Nicolet iS5, Thermo Fisher, USA). The chemical components of obtained samples were characterized by the X-ray photoelectron spectroscopy (XPS, Thermo ESCALAB 250XI). For the investigation of morphology and micro-structure, FESEM (Zeiss_Supra55) and HRTEM (Tecnai 12) were employed.

Electrochemical and Desalination Measurement

The working electrode was fabricated by blending the as-prepared active material (80 wt.%), carbon black (10 wt.%), and polyvinylidene fluoride (10 wt.%) in N-methyl-2-pyrrolidone solution to achieve uniform slurries. Subsequently, these slurries were cast onto graphite substrates and vacuum-dried overnight at 80 °C. The electrodes used for electrochemical experiments were targeted to have a mass of approximately 5 mg, a thickness of around 200 μm, and dimensions of 2×2 cm^2. All the electrochemical experiments were performed on CHI660B electrochemical workstation in a three-electrode system containing a working electrode (the fabricated electrodes), reference electrode (Ag/AgCl electrode), and counter electrode (platinum foil) in 1.0 M Na_2SO_4 solution.

The specific capacitance (C, F g^{-1}) of electrode material was calculated based on the cyclic voltammetry curves through the following formula:[2]

$$C = \int idV \, / \, 2\Delta V m v \tag{1}$$

where i, ΔV, m, and v mean the current (A), potential window (V), mass of active materials (g), and scan rate (V s^{-1}), respectively.

Desalination experiments were conducted in a batch-mode hybrid capacitive deionization (CDI) cell comprising a pair of asymmetrical electrodes, employing the prepared samples as the cathode and activated carbon (AC) as the anode, with both anion-exchange and cation-exchange membranes incorporated. The desalination electrodes were fabricated following the identical procedure employed in the electrochemical experiments. Each desalination electrode measures 6 × 6 cm^2 in size. During operation, NaCl solution was used as the target solution at 298 K which was continuously circulated in the CDI cell driven by a peristaltic pump with a flow rate of 100 mL min^{-1}. In each batch mode test, a constant voltage was supplied on the unit cell by CHI660B electrochemical workstation. The variation of NaCl concentration was detected by using the online conductivity meter (Precision and Scientific Instrument, DDS-308). The relationship between conductivity and concentration has been described in our previous works.[3, 4]

The SAC (mg g^{-1}) and corresponding SACR (mg g^{-1} min^{-1}) were defined as follows:[3]

$$SAC = (C_0 - C) \times V/m \tag{2}$$

$$SACR = SAC/t \tag{3}$$

where C_0 and C are initial and final concentrations of NaCl solution (mg L^{-1}), respectively; V refers to the volume of NaCl solution (L); m is the total mass of active material for both electrodes (g); and t is the desalination time (min).

The charge efficiency (Λ) is defined as follows:[4]

$$\Lambda = \frac{SAC \times F}{\Sigma} \tag{4}$$

where F denotes the Faraday constant (96485 C mol^{-1}). ∑ (charge, C g^{-1}) is calculated by integrating the corresponding current time curve.

Computational Methods

All the first principles calculations were performed within the framework of density functional theory (DFT) as implemented in the Vienna Ab initio Software Package (VASP 5.4.4) code. For calculation, the Perdew-Burke-Ernzerhof (PBE) generalized gradient approximation and the projected augmented wave (PAW) method were employed.[5-8] The 450 eV was set for the cutoff energy for the plane-wave basis. Besides, the Brillouin zone of the surface unit cell was sampled by Monkhorst-Pack (MP) grids.[9] For the optimization of NiHCF and Mn-NiHCF, the same k-point mesh was used. The NiHCF bulk structure was determined by a 3×3×3 Monkhorst-Pack grid. Specifically, the equilibrium lattice constants for Ni-PB bulk were found to be $a = b = c = 10.07$ Å, which were in good agreement with experimental values ($a = b = c = 10.23$ Å).[10-12] The convergence criterion for the electronic self-consistent iteration was set to 10^{-7} eV, and for force, it was set to 0.01 eV $Å^{-1}$. Prior to the calculation, the unit cell structures were optimized. The climbing image nudged elastic band (CI-NEB) method was used to confirm the transition states that possess only one imaginary frequency along the reaction coordinates.[12] The activation barrier (E_a) and reaction energy (E_r) were defined as follows: $E_a = E_{TS} - E_{IS}$, where E_{TS} represents the energy of the transition state, E_{IS} represents the energy of the initial state, and E_{FS} represents the energy of the final state.

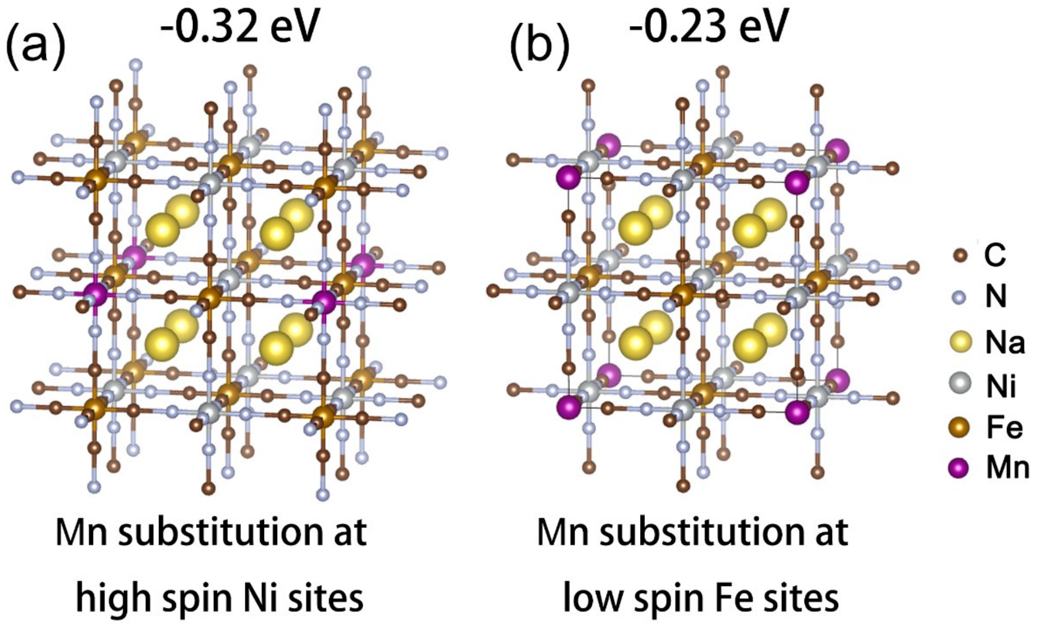

Fig. S1 Energy calculations for NiHCF with different Mn substitution sites: (a) high spin Ni sites and (b) low spin Fe sites.

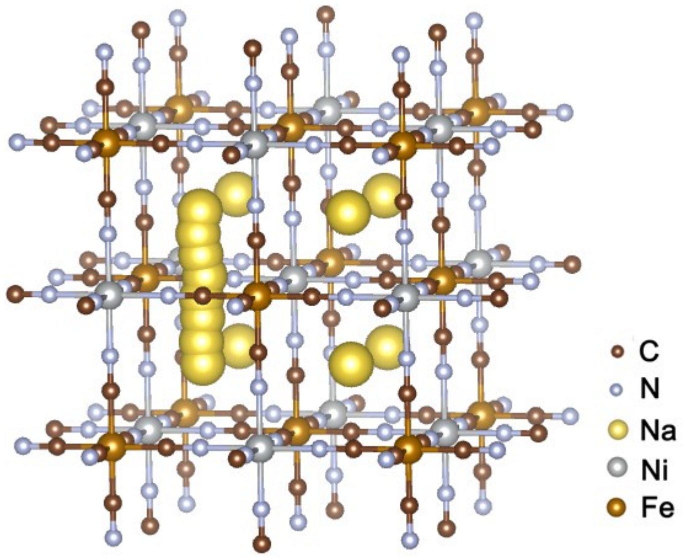

Fig. S2 Possible Na^+ diffusion pathways inside ideal NiHCF structure.

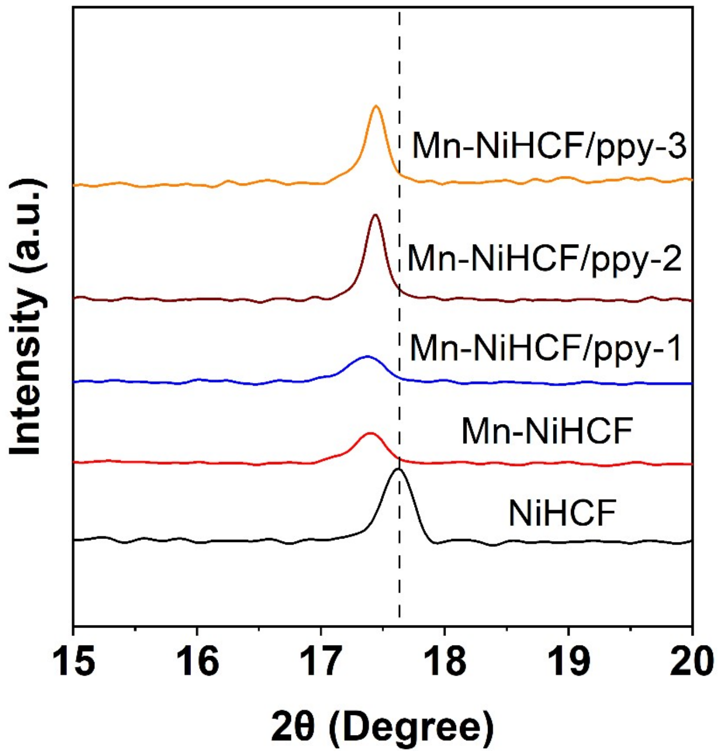

Fig. S3 Enlarged XRD patterns for NiHCF, Mn-NiHCF, Mn-NiHCF/ppy-1, Mn-NiHCF/ppy-2, and Mn-NiHCF/ppy-3 between 15 and 20°.

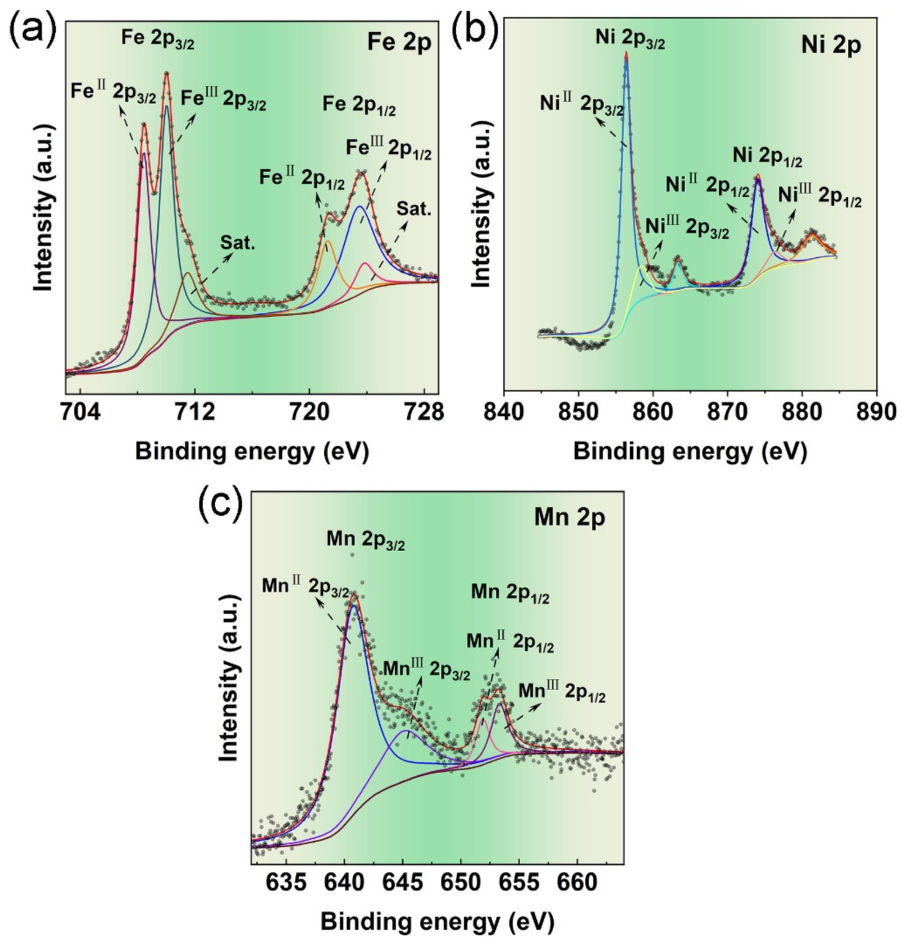

Fig. S4 High-resolution XPS spectra of (a) Fe 2p, (b) Ni 2p, and (c) Mn 2p for Mn-NiHCF/ppy-2.

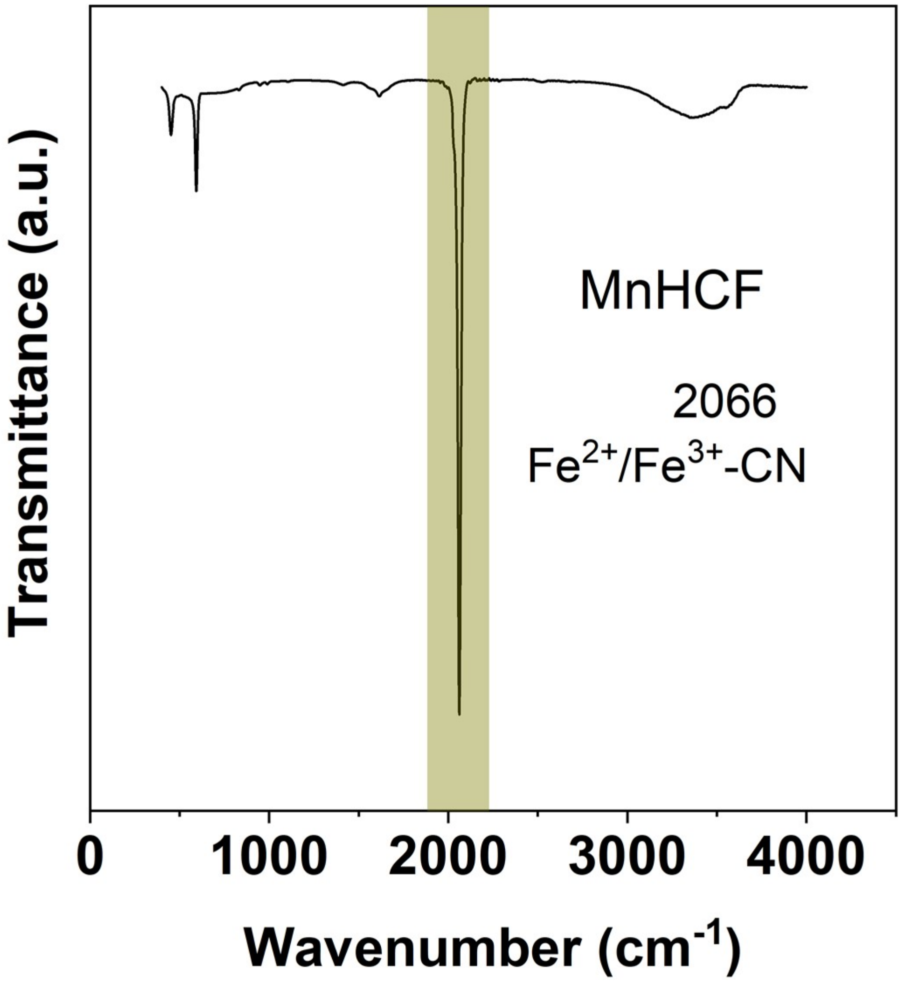

Fig. S5 The FTIR spectrum of pure MnHCF.

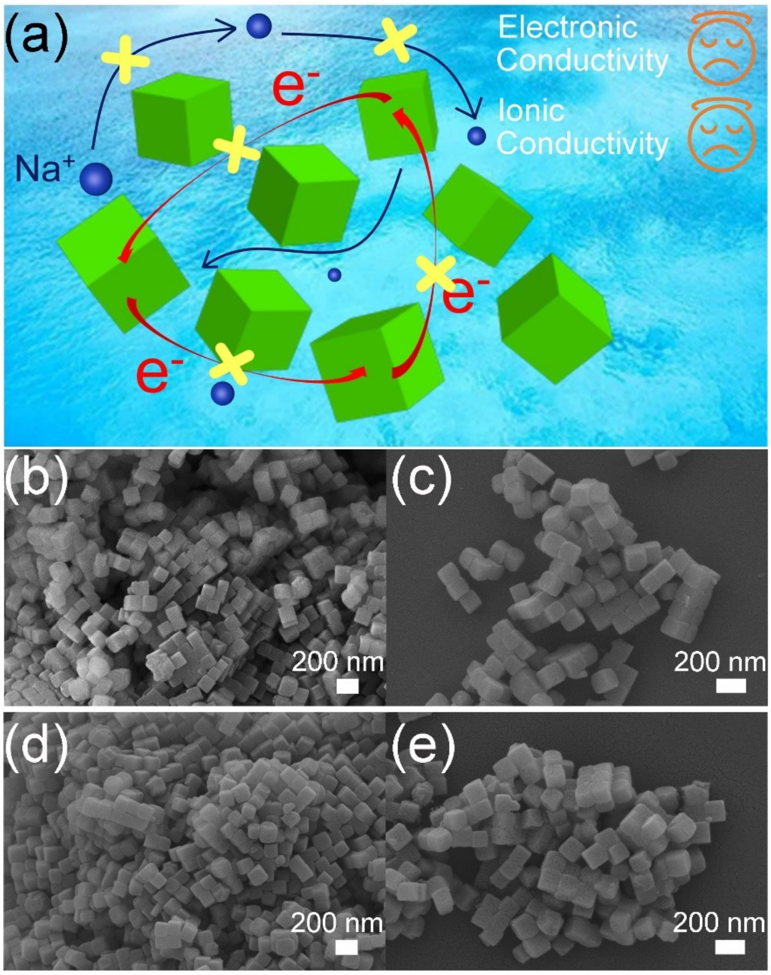

Fig. S6 (a) Illustration of the ion/electron migration pathway and (b-e) FESEM images with different magnifications of (b, c) NiHCF and (d, e) Mn-NiHCF.

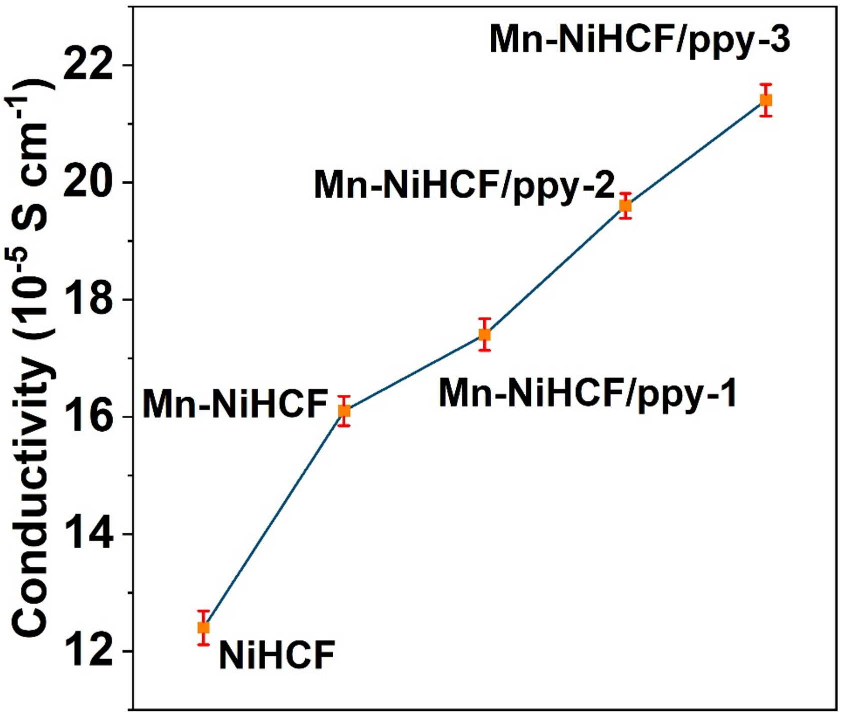

Fig. S7 Electronic conductivities of the as-prepared NiHCF, Mn-NiHCF, Mn-NiHCF/ppy-1, Mn-NiHCF/ppy-2, and Mn-NiHCF/ppy-3 obtained from four-probe measurements.

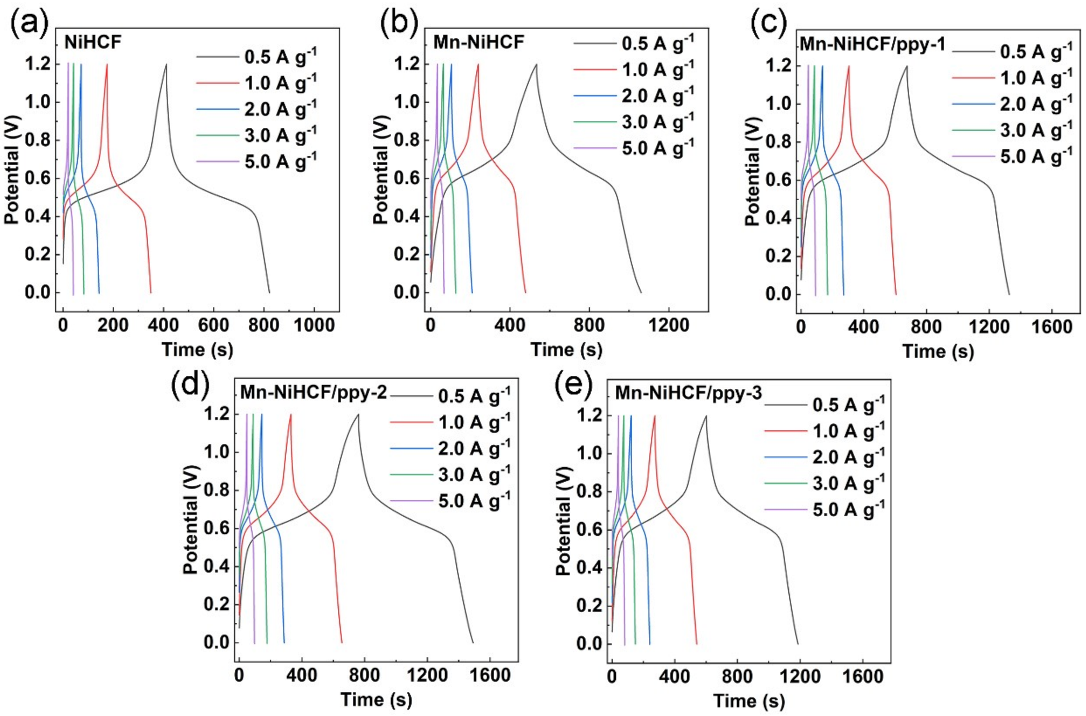

Fig. S8 Galvanostatic discharge/charge profiles of (a) NiHCF, (b) Mn-NiHCF, (c) Mn-NiHCF/ppy-1, (d) Mn-NiHCF/ppy-2, and (e) Mn-NiHCF/ppy-3 at various current densities.

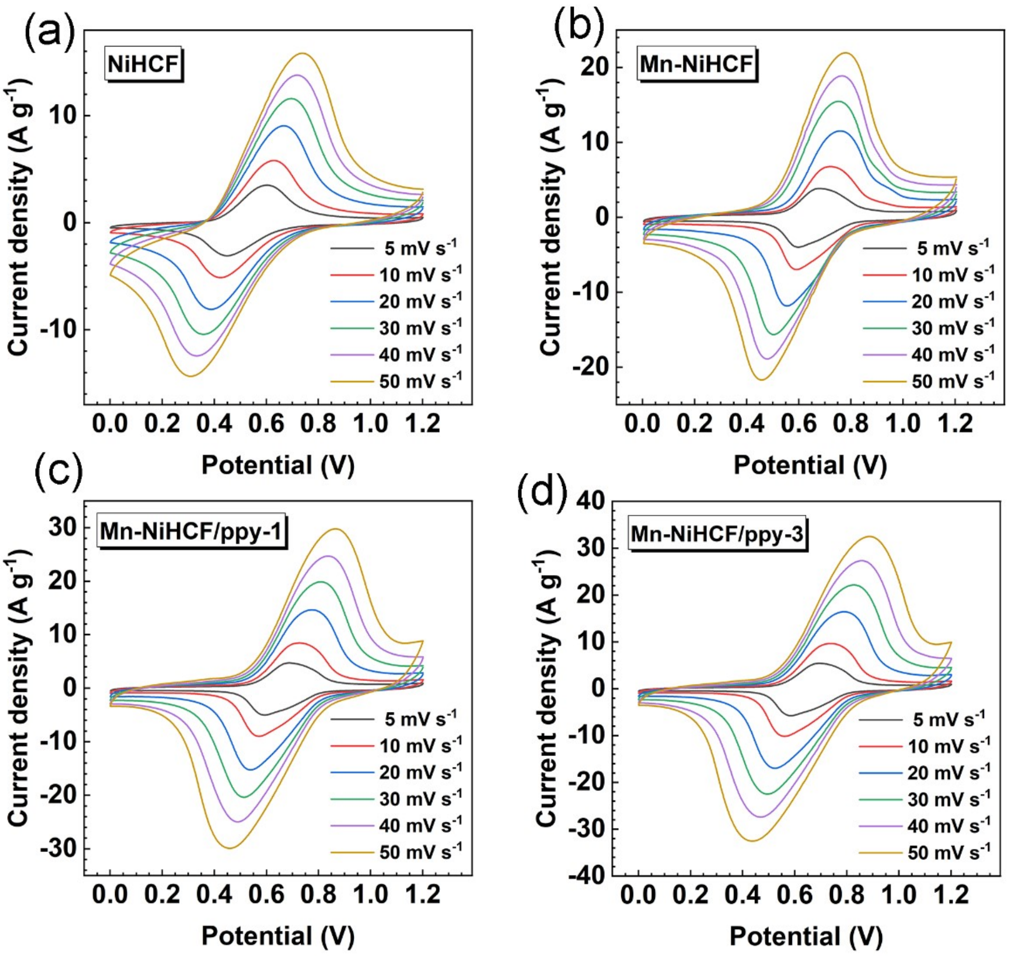

Fig. S9 CV curves at various scan rates of (a) NiHCF, (b) Mn-NiHCF, (c) Mn- NiHCF/ppy-1, and (d) Mn-NiHCF/ppy-3.

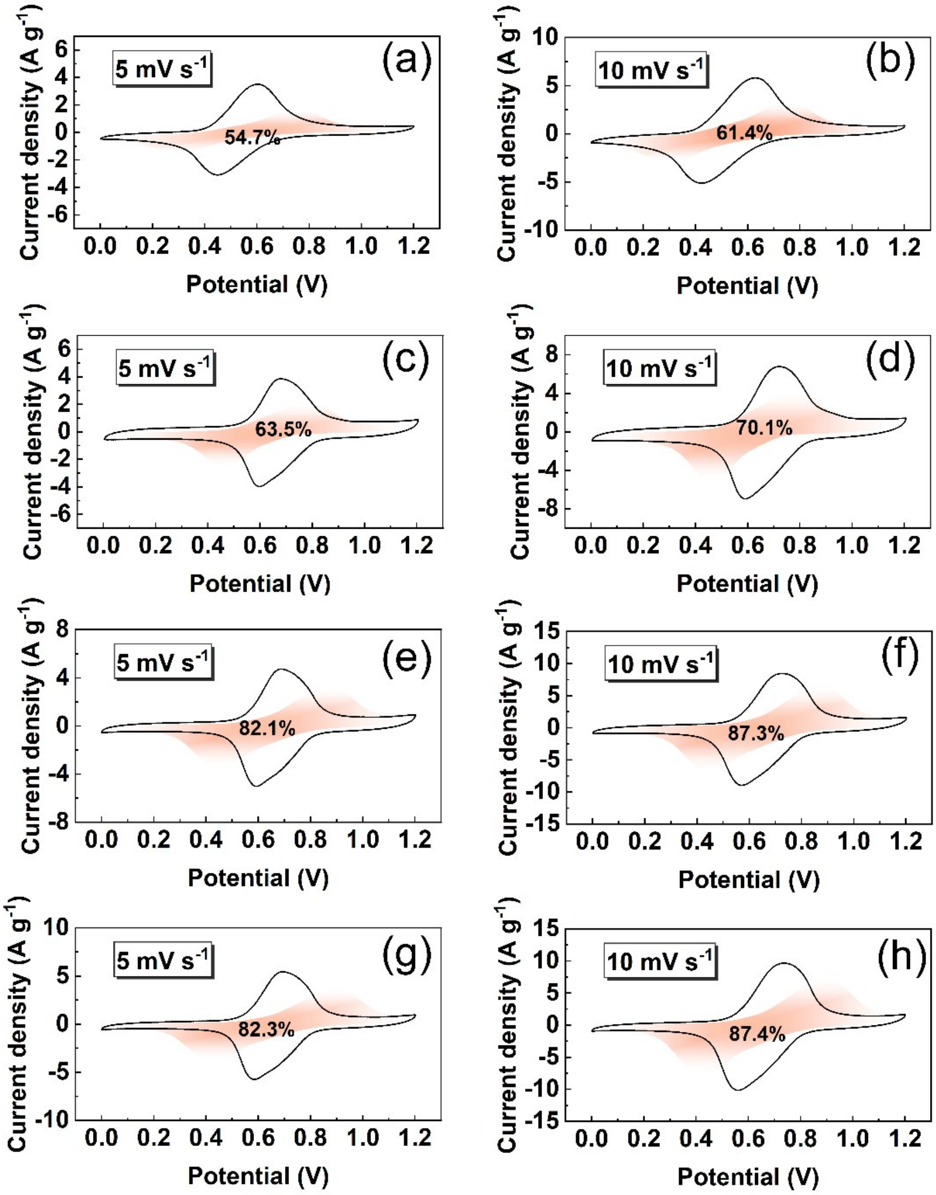

Fig. S10 Capacitive contributions of (a, b) NiHCF, (c, d) Mn-NiHCF, (e, f) Mn-NiHCF/ppy-1, and (g, h) Mn-NiHCF/ppy-3 at 5.0 and 10.0 mV s^{-1}.

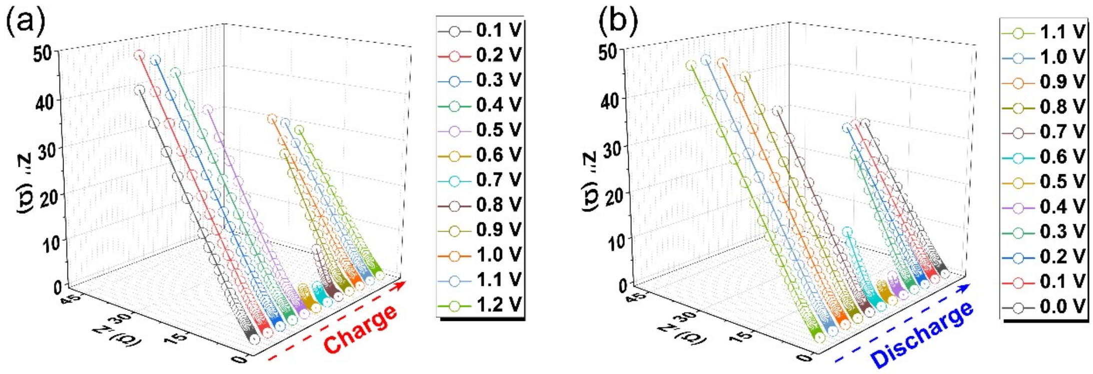

Fig. S11 In-situ EIS measurements of Mn-NiHCF/ppy-2 during (a) charging and (b) discharging processes.

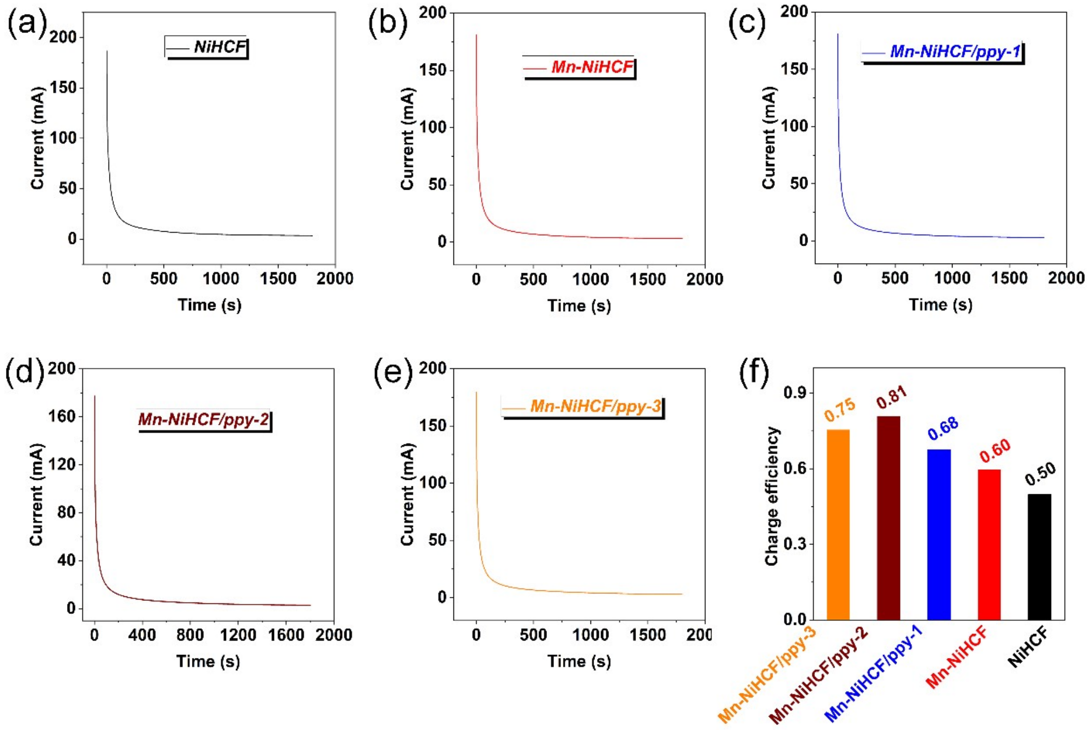

Fig. S12 Current responses of (a) NiHCF, (b) Mn-NiHCF, (c) Mn-NiHCF/ppy-1, (d) Mn-NiHCF/ppy-2, (e) Mn-NiHCF/ppy-3 and (f) corresponding charge efficiencies.

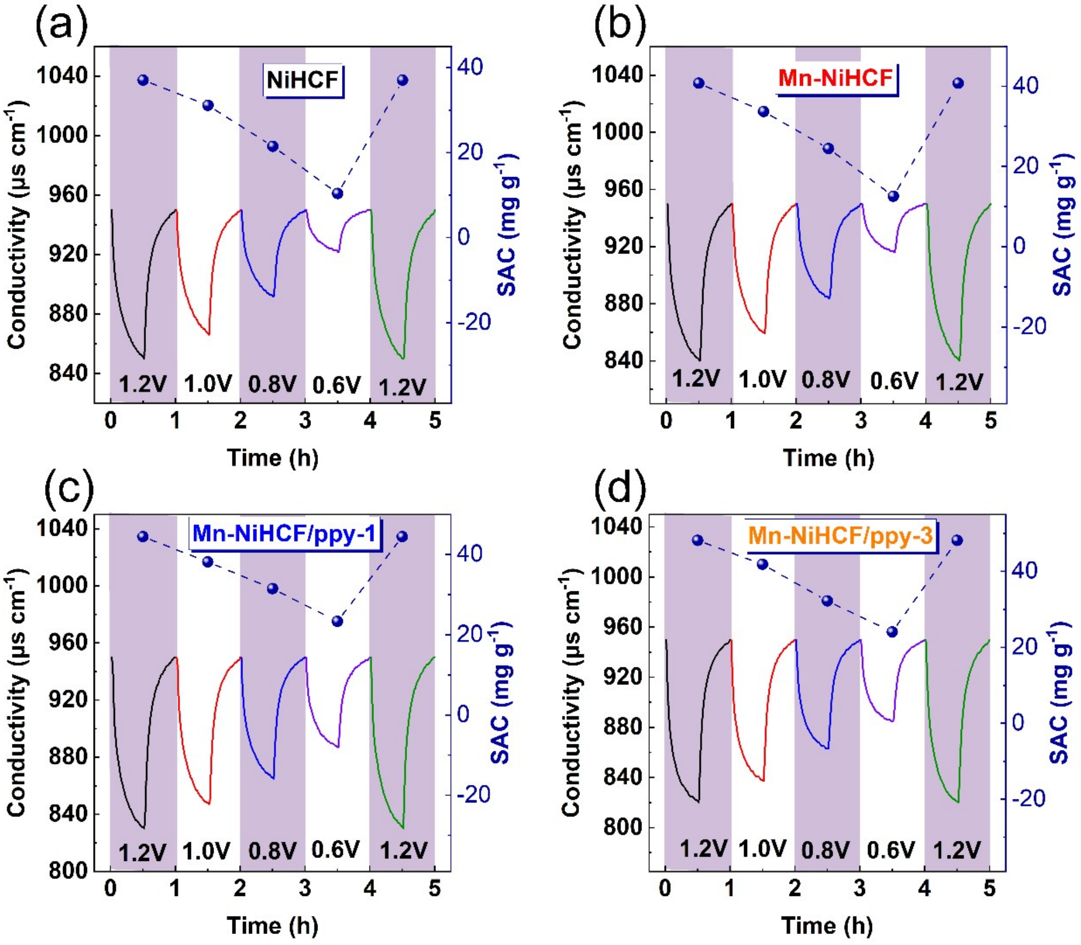

Fig. S13 Conductivity versus time profiles and SACs in CDI cell with the (a) NiHCF, (b) Mn-NiHCF, (c) Mn-NiHCF/ppy-1 and (d) Mn-NiHCF/ppy-3 electrodes in 500 mg L^{-1} NaCl solution at different voltages.

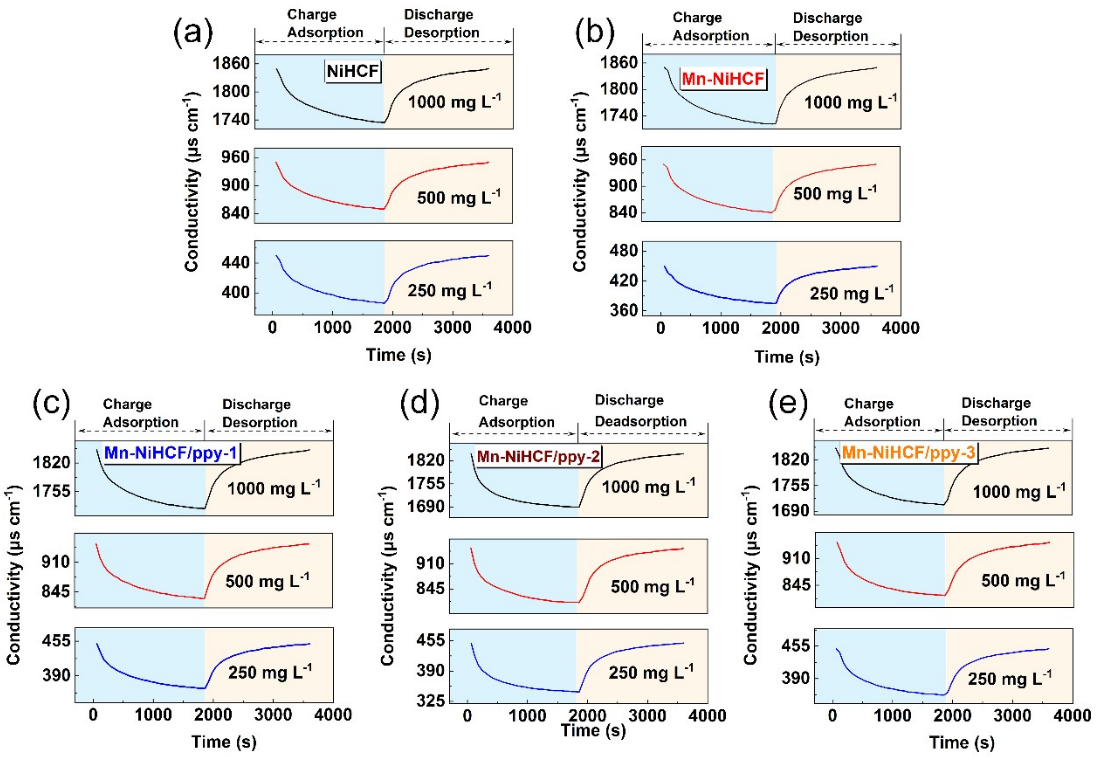

Fig. S14 Conductivity versus time profiles and SACs in CDI cell with (a) NiHCF, (b) Mn-NiHCF, (c) Mn-NiHCF/ppy-1, (d) Mn-NiHCF/ppy-2, and (e) Mn-NiHCF/ppy-3 electrodes at 1.2 V in NaCl solution with various concentrations.

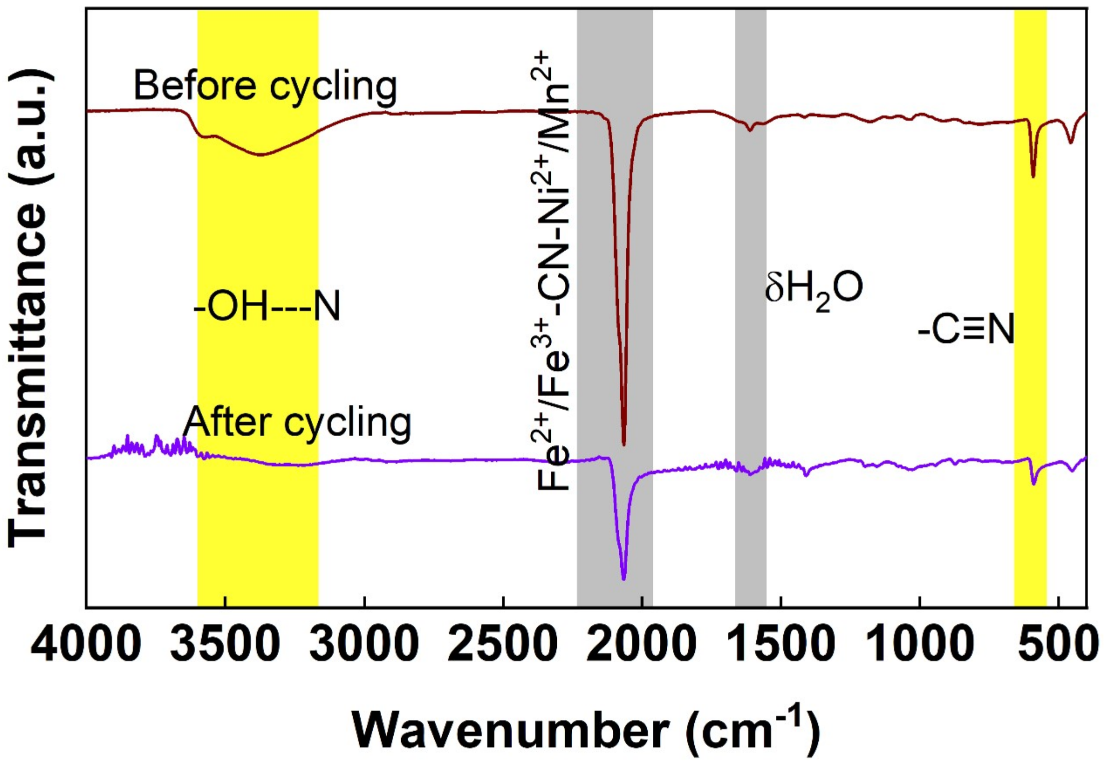

Fig. S15 Comparison of FTIR spectra of the Mn-NiHCF/ppy-2 before cycling and after 50 cycles.

Table S1 Sodium ion diffusion coefficient of as-prepared five samples.

Samples	$I_p/v^{1/2}$ (Red)	Coefficient (Red, $cm^2\ s^{-1}$)	$I_p/v^{1/2}$ (Ox)	Coefficient (Ox, $cm^2\ s^{-1}$)	Coefficient (Average, $cm^2\ s^{-1}$)
NiHCF	-2.19	6.82×10^{-11}	2.52	2.52×10^{-11}	7.93×10^{-11}
Mn-NiHCF	-3.20	1.45×10^{-10}	3.76	2.01×10^{-10}	1.73×10^{-10}
Mn-NiHCF/ppy-1	-5.10	3.70×10^{-10}	5.12	3.73×10^{-10}	3.71×10^{-10}
Mn-NiHCF/ppy-2	-6.35	5.74×10^{-10}	6.37	5.77×10^{-10}	5.76×10^{-10}
Mn-NiHCF/ppy-3	-5.47	4.26×10^{-10}	5.63	4.51×10^{-10}	4.38×10^{-10}

Table S2 SACs of the as-prepared samples at various voltages.

Sample	SAC (mg g^{-1})			
	0.6 V	0.8 V	1.0 V	1.2 V
NiHCF	10.3	21.4	31.1	37.0
Mn-NiHCF	12.5	24.2	33.7	40.7
Mn-NiHCF/ppy-1	23.3	31.4	38.1	44.4
Mn-NiHCF/ppy-2	25.2	35.1	43.7	52.2
Mn-NiHCF/ppy-3	24.0	32.2	41.8	48.1

Table S3 SACs of the as-prepared five samples at various saline concentrations.

Sample	SAC (mg g^{-1})		
	250 mg L^{-1}	500 mg L^{-1}	1000 mg L^{-1}
NiHCF	23.7	37.0	42.9
Mn-NiHCF	28.1	40.7	47.7
Mn-NiHCF/ppy-1	31.1	44.4	51.8
Mn-NiHCF/ppy-2	38.8	51.8	55.5
Mn-NiHCF/ppy-3	34.4	48.1	53.3

REFERENCES (1-8)

1. X. Xu, J. Tang, Qian, H. Qian, S. Hou, Y. Bando, M. S. A. Hossain, L. Pan and Y. Yamauchi, *ACS Appl. Mater. Interfaces*, 2017, **9**, 38737-38744.

2. X. Liu, X. Xu, X. Xuan, W. Xia, G. Feng, S. Zhang, Z. G. Wu, B. Zhong, X. Guo, K. Xie and Y. Yamauchi, *J. Am. Chem. Soc*., 2023, **145**, 9242-9253.

3. Y. Zhang, J. Wu, S. Zhang, N. Shang, X. Zhao, S. M. Alshehri, T. Ahamad, Y.; Xu, X. Yamauchi and Y. Bando, *Nano Energy*, 2022, **97**, 107146.

4. J. Guo, X. Xu, J. P. Hill, L. Wang, J. Dang, Y. Kang, Y. Li, W. Guan and Y. Yamauchi, *Chem. Sci.*, 2021, **12**, 10334-10340.

5. J. P. Perdew, K. Burke and M. Ernzerhof, *Phys. Rev. Lett*., 1996, **77**, 3865-3868.

6. B. Hammer, L. B. Hansen and J. K. Nørskov, *Phys. Rev. B*., 1999, **59**, 7413-7421.

7. P. E. Blöchl, *Phys. Rev. B*., 1994, **50**, 17953-17979.

8. G. Kresse and D. Joubert, *Phys. Rev. B*., 1999, **59**, 1758-1775.

9. H. J. Monkhorst and J. D. Pack, *Phys. Rev. B*., 1976, **13**, 5188-5192.

10. A. J. Fernández-Roperoa, M. J. Piernas-Muñoza, E. Castillo-Martíneza, T. Rojo and M. Casas-Cabanas, *Electrochim. Acta*., 2016, **210**, 352-357.

11. G. Henkelman and H. Jonsson, *J. Chem. Phys*., 2000, **113**, 9978-9985.

12. G. Henkelman, B. P. Uberuaga and H. Jónsson, *J. Chem. Phys*., 2000, **113**, 9901-9904.
